# Attosecond high-harmonic interferometry probes orbital- and band-dependent dipole phase in magnesium oxide

**DOI:** 10.1126/sciadv.aeb4109

**Published:** 2026-05-01

**Authors:** Nataliia Kuzkova, Pieter J. van Essen, Roy van der Linden, Brian de Keijzer, Rui E. F. Silva, Álvaro Jiménez Galán, Peter M. Kraus

**Affiliations:** ^1^ Advanced Research Center for Nanolithography (ARCNL), Science Park 106, 1098 XG Amsterdam, Netherlands.; ^2^ Department of Physics and Astronomy, LaserLaB, Vrije Universiteit (VU), De Boelelaan 1105, 1081 HV Amsterdam, Netherlands.; ^3^ Instituto de Ciencia de Materiales de Madrid, Consejo Superior de Investigaciones Científicas (ICMM-CSIC), Sor Juana Inés de la Cruz 3, 28049 Madrid, Spain.; ^4^ Max-Born-Institute for Nonlinear Optics and Short Pulse Spectroscopy (MBI), Max-Born-Strasse 2A, D-12489 Berlin, Germany.

## Abstract

Control over the spatial coherence, wavefront, and focusability of emitted light relies on understanding the intrinsic phase of the emission process, and vice versa; measuring the phase can provide insights about microscopic generation mechanisms. Using attosecond interferometry with phase-locked extreme ultraviolet (XUV) pulses, we directly measure the phase differences of XUV pulses emitted from two separate foci on the same solid sample that generate high harmonics at different driving intensities, allowing us to assess the intensity- and frequency-dependent dipole phase. Our results are supported by analytical, two-band, and full-band numerical theoretical models. The analytical approach benefits future solid-state high-harmonic generation studies, while the full numerical model details orbital- and band-resolved current contributions to the dipole phase. This research delivers combined quantitative measurement and rigorous theoretical description of the harmonic emission phase in solids.

## Teaser

Unlocking solid-state high-harmonic phase with attosecond interferometry reveals insights into microscopic emission mechanisms.

## INTRODUCTION

High-harmonic generation (HHG) from solids under strong laser fields presents a compelling frontier in nonlinear optics (*1*–*3*), unlocking possibilities for ultrafast spectroscopy and attosecond science. Recent advancements in this field highlight the potential of solid-state HHG as a spectroscopy (*4*–*6*) and optically controlled superresolution microscopy technique (*7*, *8*), as well as a compact source of extreme ultraviolet (XUV) radiation, characterized by a high degree of temporal and spatial coherence. This coherence is essential for innovative applications such as lensless diffractive imaging and precision metrology at the nanoscale (*9*–*11*), fueling the pursuit of all-solid-state HHG sources capable of generating coherent and focused XUV light (*12*, *13*). Achieving efficient HHG-based sources and optimal HHG beam focusability (*14*, *15*) requires careful consideration of the spatial coherence of emitted harmonics, which is fundamentally governed by the phase and divergence of the resulting XUV wavefront. The phase of high-harmonic emission, determined by the dipole phase, strongly depends on the intensity and frequency of the fundamental laser field (*16*, *17*). In the following, we refer to the intensity dependence of the emission phase, which is the subject of the present manuscript, as dipole phase. The spectral phase, which is not studied here, is referred to as an attochirp. As electrons in the atoms or molecules of the generating HHG medium interact with the intense laser field, they traverse various electronic quantum paths, ultimately recombining with their parent ions to emit XUV photons at harmonics of the driving frequency. In gases, this process is well understood through a semiclassical framework (*18*–*21*), where two energetically degenerate pathways, known as the short and long electron trajectories, play a pivotal role in determining the dipole phase of emitted harmonics. There, the characteristics of the driving laser beam—such as wavelength, peak intensity, polarization state, and temporal profile—primarily shape the dipole phase, rather than the specific atomic species being used (*14*, *22*–*26*). In solid-state HHG, however, the behavior is distinct from that in gases, as solids have a well-defined electronic band structure that substantially affects electron dynamics in the presence of strong laser fields (*27*–*29*). The microscopic response of HHG in solids shows that the laser field–induced transitions of electrons between the valence band (VB) and the conduction band (CB) introduce two principal contributions to the dipole phase: interband and intraband dynamics (*30*–*33*). Interband dynamics involve polarization effects that drive electronic excitation and electron-hole recombination, while intraband dynamics pertain to the oscillatory motion of tunnel-ionized electrons within a single band. Both contributions interact intricately with the laser field parameters and the material’s electronic properties, leading to multifaceted phase characteristics in solid-state HHG responses.

Despite its importance, only a few recent experimental studies have focused on examining the dipole phase in solid-state HHG (*34*–*36*). While linear relationships between the phase of solid-state HHG and intensity—similar to gas-phase HHG—have been established (*35*), a clear link to a microscopic model via theory beyond few-level simulations is missing. In light of this, the present study aims to close this knowledge gap by investigating the dipole phase in the solid-state HHG, bolstered by robust theoretical calculations. The complexities inherent to measuring and manipulating the dipole phase in solid-state HHG pose substantial challenges, demanding meticulously designed experiments and sophisticated theoretical modeling. However, recent advancements in XUV interferometry techniques have advanced our ability to probe HHG phenomena at the atomic level, both in gases and solids (*35*–*40*). XUV interferometry offers a unique and powerful approach for examining the dipole phase and its contribution to high-harmonic emission. By interfering two XUV pulses, the phase of the emitted harmonics can be directly quantified and obtained. Nevertheless, conducting XUV interferometry experiments often comes with challenges related to achieving and maintaining high stability and intrinsic interferometric delay precision at the subwavelength level, particularly in terms of phase control. The widely used XUV interferometers, such as those using Wollaston prisms (*41*), curved two-segment mirrors (*40*), or Mach-Zehnder (*42*) or Michelson-type (*43*) designs, often experience changes in the different optical paths because of misalignments, thermal expansion, or mechanical instabilities, resulting in phase variations and reduced stability. Thus, these interferometers require active stabilization of optical components and feedback loops to achieve fine control over the phase (*44*, *45*).

In this work, we use an ultrastable birefringent common-path interferometer that incorporates two delayed, phase-locked collinear replicas of an input ultrashort near-infrared (NIR) laser pulse to generate two well-controlled XUV sources with attosecond precision. The interferometer design draws inspiration from TWINS (translating wedge–based identical pulses encoding system) (*46*) and has been successfully implemented in multiple spectral regions (*46*–*50*). Here, we leverage XUV interferometry to assess the dipole phase of high harmonics generated from bulk magnesium oxide (MgO) crystalline solid. We resolve the harmonic phase shifts by measuring the corresponding interference fringes in the far field, varying the relative peak intensities of two spatially separated fundamental 800-nm beams focused on the MgO target. This methodology allows us to directly probe the intensity- and frequency-dependent relative dipole phase of high harmonics in the solid. Furthermore, through the dipole phase measurements, we reveal that our interferometric setup is sensitive to the nonlinear phase shift, namely the B-integral, accumulated as the intense laser beam travels through a nonlinear medium. We find that the contribution from this intrinsic parameter, reflecting the degree to which laser intensity modifies the refractive index of the material, can be individually identified and subsequently subtracted from the measured harmonic emission phase while maintaining the same experimental conditions. Thus, this study introduces previously unknown quantitative measurements of the dipole phase and nonlinear effects occurring in real time in solid-state HHG, achieved using an ultrastable XUV interferometer. To evaluate the stability of our interferometer and guarantee the accurate synchronization of the generated harmonics with the fundamental frequency, we perform temporal XUV interferometric measurements, which involve the interference of two time-delayed coherent XUV fields. By controlling their relative time delay with attosecond-level precision, we can accurately determine the time-dependent phase delays of individual harmonics within one optical cycle (1 OC) of an 800-nm driver. We implement several theoretical models and simulations to assist with the analysis of the intensity-dependent experimental data and clarify the physical origins of the harmonic dipole phase in bulk MgO. First, an analytical semiclassical HHG model is applied to elucidate the similarities and differences in harmonic dipole phase between gases and solids, given that the interband mechanism in solids exhibits a recollision-like behavior akin to that of gaseous atoms or molecules. Next, numerical simulations based on the semiconductor Bloch equations (SBEs) are performed for the two-band system, incorporating the material’s electronic band structure and the interplay between interband and intraband contributions to the dipole phase. Last, we implement an orbital-based framework in the Wannier basis for the multiband system using numerical SBE calculations, providing a clear picture of how different orbitals contribute to the overall harmonic dipole phase and shedding light on the underlying physics of the system.

## RESULTS

### Spectrally resolved XUV interferometry

**Fig. 1.**
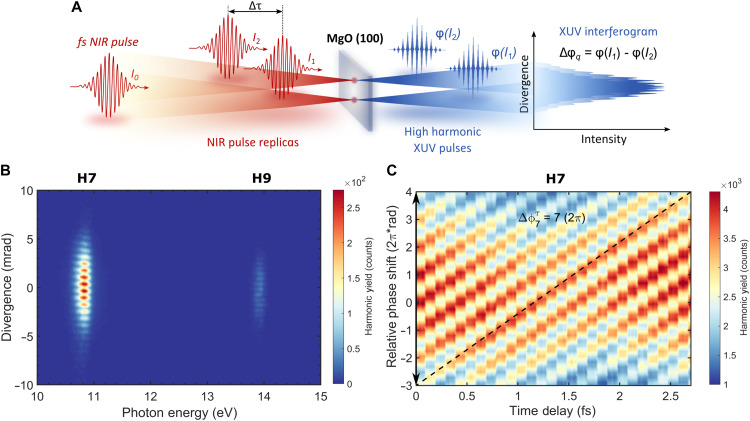
Spectrally resolved XUV interferometry of MgO solid. (**A**) Concept of the XUV interferometric experiment for measuring the phase of high-harmonic emission in solids. (**B**) Far-field XUV interferograms obtained from MgO, presented with the intensities of the NIR foci set to be identical (6 TW cm^−2^). The harmonic orders (H7 and H9) refer to the odd multiples of the 800-nm driver. (**C**) Time-dependent relative phase shift, $\Delta \phi_{q}^{\tau }$, in MgO for harmonic order *q* = 7, as obtained from the temporal XUV interferograms. The relative fringe shift of H7 is shown with a dashed line. The extracted $\Delta \phi_{7}^{\tau }$ value over 1 OC of the 800-nm laser field (τ_800_ = 2.66 fs) is indicated by a black arrow and is depicted in the figure.

The spectrally resolved XUV interferometric experiment in bulk MgO is depicted in Fig. 1A. An input femtosecond NIR pulse with the intensity *I*_0_ is transformed into two driving NIR pulse replicas by a birefringent common-path interferometer, which are subsequently focused into a solid HHG medium to generate XUV pulses while maintaining a vertical separation between their focal points on the target. Following the generation of two spatially separated XUV sources from these foci, the emitted light is spectrally dispersed in the horizontal direction and spatially diverged vertically, yielding a spatiospectral image with interference horizontal fringes of high-harmonic emissions captured in the far-field. The relative dipole phase of high harmonics, Δϕ*_q_*, is assessed by varying the relative peak intensities of the two pulse replicas (*I*_1_ and *I*_2_) and measuring the intensity-induced fringe shifts [ϕ(*I*_2_) and ϕ(*I*_2_)] of the individual harmonics *q*. The interferometer’s reliability and precise synchronization of the produced harmonics with the fundamental frequency are evaluated by measuring the relative phase delay of high harmonics as a function of time (Δτ). This process entails examining the interference fringe shifts between two coherent XUV fields that are time delayed while ensuring that the intensity distribution remains equal across the NIR foci (i.e., *I*_1_ = *I*_2_).

Figure 1B exemplarily shows the far-field XUV interferograms obtained from the bulk MgO with both driving NIR beams having matched intensities at their spatiotemporal peak (6 TW cm^−2^ at each focus). The odd-order harmonics such as harmonic 7 (H7, 10.8 eV) and harmonic 9 (H9, 13.9 eV) were generated with an 800-nm laser driver following the experimental conditions outlined in Materials and Methods. The fringe patterns observed in the far-field interferograms on the detection plane, which are proportional to the separation distance of the NIR foci, were chosen to effectively resolve phase shifts in high harmonics while avoiding oscillations at the fundamental frequency. Figure 1C shows the two-dimensional (2D) color map representing the time-dependent relative phase shift of H7 in MgO, recorded as a function of the time delay between the NIR pulse replicas. The Δτ was varied by translating one of the birefringent wedges of the interferometer transversely over a Δ*x* = 30 μm range with a step size of 0.2 μm corresponding to a scanned Δτ = 2.96 fs range and a minimum step motion of 20 as. This was sufficient to follow 1 OC of the 800-nm driving wavelength, τ_800_ = 2.66 fs, with a high spectral resolution. As a result, the time-dependent relative phase delays of the generated harmonics were defined with a sub–20-as accuracy, providing detailed insights into their temporal evolution. By measuring the displacement of the fringes over time for H7 and H9, we validated the robustness of our common-path interferometer and the precise synchronization of harmonics with the NIR driver, ensuring subcycle phase locking. Given that the two foci are identical, the dipole phase term (which relies on the driving field intensity) is the same for both, and therefore, it does not affect the temporal harmonic phase difference (*20*). In Fig. 1C, the relative phase shift of H7 over the τ_800_ range is fitted with a linear function (dashed line). The time-dependent relative phase of the harmonic field is determined within a 2.7-fs range (black arrow). Here, the maxima and minima of the fringe pattern indicate a (2π)·rad phase shift that represents the distance between two peaks and their relative displacement. The time delay affects the phase of the generated harmonics in a linear manner, expressed as follows (*37*): $\Delta \phi_{q}^{\tau }=\tau_{800}\cdot q$, where $\Delta \phi_{q}^{\tau }$ represents the relative phase shift associated with the *q*-th harmonic order. Analysis of the experimental data, as illustrated in Fig. 1C, shows that the extracted phase shift for H7 is $\Delta \phi_{7}^{\tau }$ = 7 (2π)·rad, which corresponds exactly to the harmonic order *q* = 7. Furthermore, temporal XUV interferometric results for H9 corroborate that this linear dependence holds for higher-harmonic orders (fig. S9). These findings underscore the exceptional resolution and sensitivity to the harmonic phase, achieved through the XUV interferometric approach used here. Moreover, the absence of any fringe oscillation component corresponding to the periodicity of the fundamental (i.e., with a periodicity of 1 OC) indicates that the two HHG sources are well separated in the near-field and do not overlap, which would otherwise obscure the subsequent extraction of the dipole phase.

### Harmonic relative dipole phase and nonlinear optical effects

The intensity-induced relative dipole phase of high harmonics in MgO was investigated by changing the relative peak intensities between the NIR foci. The intensities of the NIR beams, *I*_1_ and *I*_2_, were linearly varied over the Δ*I*/*I*_0_ = ±1 range by rotating a half-wave plate (HWP) positioned in front of the interferometer (fig. S1), where *I*_0_ is the sum of the input intensities (*I*_1_ + *I*_2_) and Δ*I* is their difference (*I*_1_ − *I*_2_). A value of ±1 means that all the intensity is placed into a single beam (either *I*_1_ = 1 and *I*_2_ = 0 or *I*_1_ = 0 and *I*_2_ = 1), resulting in no interference, while a value of 0 represents equal intensity between the two focal points (*I*_1_ = *I*_2_). The far-field XUV interferograms of the spectrally dispersed high harmonics were recorded as a function of the HWP rotational angle, $\theta_{\text{HWP}}$, associated with Δ*I*/*I*_0_. The ±1 positions represent $\theta_{\text{HWP}}$ angles of 0° (0 rad) and 45° (π/4 rad), which corresponds to a 90° rotation in the laser polarization angle. The total peak intensity in one beam was estimated to be ~12 TW cm^−2^. With equal intensity at both focal points, i.e., Δ*I*/*I*_0_ = 0, the peak intensity was 6 TW cm^−2^ ($\theta_{\text{HWP}}$ = 22.5°). During the experiments, the interference fringes were distinctly observed within a Δ*I*/*I*_0_ = ±0.5 range, corresponding to $\theta_{\text{HWP}}$ scan angles from 15° to 30°, or equivalently ±7.5° around the zero Δ*I*/*I*_0_ position. This angular range matched variations in peak driving intensities from 3 to 9 TW cm^−2^ in one arm (Δ*I*/*I*_0_ = −0.5) and inversely in the other arm (Δ*I*/*I*_0_ = 0.5). We selected this range to analyze the intensity-induced relative fringe shifts throughout all measurements. Further details on the NIR beam characterization and intensity calibration procedures are provided in the Supplementary Materials.

**Fig. 2.**
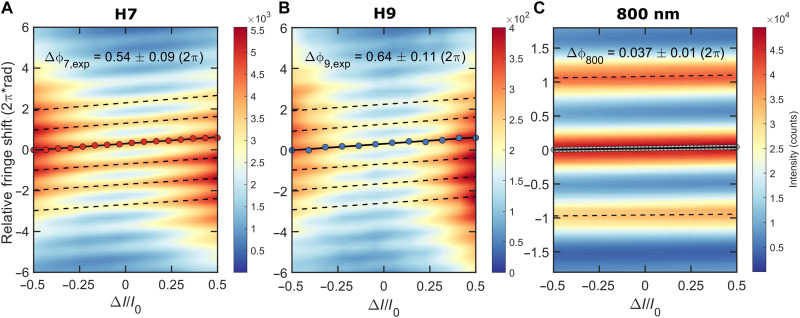
Intensity-induced relative fringe shifts for the harmonic and fundamental 800-nm emissions from MgO. The relative fringe shifts, $\Delta \phi_{q,\text{exp}}$, in MgO for H7 (**A**) and H9 (**B**) measured along the Γ-X (Mg─O bond) direction as a function of the normalized intensity difference of the NIR pulse pair Δ*I*/*I*_0_ (*I*_0_ = 12 TW cm^−2^). The Δ*I*/*I*_0_ = ±0.5 range corresponds to variations in NIR focus peak intensities from 3 to 9 TW cm^−2^ in one arm and inversely in the other; equal intensities (6 TW cm^−2^) occur at Δ*I*/*I*_0_ = 0. (**C**) Accumulated nonlinear phase shift, Δϕ_800_ (B-integral), in MgO at 800 nm, measured over the same intensity range. The $\Delta \phi_{q,\text{exp}}$ and Δϕ_800_ values, depicted in the figures, were determined for the local fringe maxima of H7 (red dots), H9 (blue dots), and fundamental (gray dots) through linear fits of the fringe shifts (dashed lines) over the Δ*I*/*I*_0_ = ±0.5 range. The $\Delta \phi_{q,\text{exp}}$ values (corrected for B-integral contributions for harmonic orders *q* = 7 and 9 and expressed in radians) are presented in Table 1.

Figure 2 (A and B) shows the 2D color maps of the intensity-induced relative fringe shifts associated with a change in the dipole phase, $\Delta \phi_{q,\text{exp}}$, for H7 (Fig. 2A) and H9 (Fig. 2B) in the bulk MgO recorded as a function of the varying relative peak intensities of the NIR pulse replicas. In the intensity-dependent measurements, the interaction of intense 800-nm laser pulses with the MgO solid resulted in an additional nonlinear response from the material. Given that the refractive index of the medium varies with intensity because of the optical Kerr effect (*51*), it leads to a nonlinear modulation of the refractive index in MgO. This modulation induces a nonlinear phase shift in the light wavefront, known as the B-integral. Thus, the B-integral is a critical parameter in this context. In our interferometric arrangement, we took advantage of the setup’s sensitivity to phase changes to measure the intensity-dependent nonlinear phase shift in the solid sample (fig. S13). Comprehensive details regarding the B-integral characterization using common-path interferometry are provided in (*52*). Figure 2C shows the 2D color map of the relative nonlinear phase shift, Δϕ_800_, accumulated by the 800-nm driver in MgO within the same measured intensity range, as displayed in Fig. 2 (A and B). By analyzing the corresponding interference patterns, we could gain insights into the nonlinear phase shift of the investigated medium and therefore deduce the B-integral contribution from the XUV measurements. In Fig. 2, the $\Delta \phi_{q,\text{exp}}$ and Δϕ_800_ values were determined from linear fits to the fringe shifts and the associated *y*-axis intercept over the Δ*I*/*I*_0_ = ±0.5 change in peak intensity range. For the XUV data, the extracted $\Delta \phi_{q,\text{exp}}$ values for H7 and H9 are 0.54 ± 0.09 (2π)·rad and 0.64 ± 0.11 (2π)·rad, respectively. For the 800-nm data, the Δϕ_800_ value of the accumulated B-integral in the MgO sample is determined to be 0.037 ± 0.01 (2π)·rad. Taking into account the harmonic order *q* = 7 and 9, the total B-integral contribution to the XUV phase, $\Delta \phi_{q,B}$, can be defined as $\Delta \phi_{q,B}=\Delta \phi_{800}\cdot q$. Thus, the cumulative nonlinear phase shifts in the 100-μm-thick MgO crystalline solid are found to be $\Delta \phi_{7,B}$ = 0.26 ± 0.07 (2π)·rad and $\Delta \phi_{9,B}$ = 0.33 ± 0.09 (2π)·rad. By subtracting the $\Delta \phi_{7,B}$ and $\Delta \phi_{9,B}$ contributions of the B-integral from the $\Delta \phi_{7,\text{exp}}$ and $\Delta \phi_{9,\text{exp}}$ intensity-induced harmonic phase fringe shifts, we can derive the experimental relative dipole phase in radians as $\Delta \phi_{q}=\Delta \phi_{q,\text{exp}}-\Delta \phi_{q,B}$. As a result, the obtained $\Delta \phi_{7}$ and $\Delta \phi_{9}$ values are 3.52 ± 1.01 and 3.90 ± 1.26 rad, respectively. In line with the previously reported study by Lu *et al.* (*35*), the relative dipole phase in MgO increases linearly with intensity when examining XUV emission from H7 to H9 over the same range. Nevertheless, our findings go beyond those of previous results (*35*), experimentally proving that the nonlinear phase accumulated by the intense 800-nm pulses in the sample in a transmission geometry is markedly substantial—nearly two-thirds of the total intensity–induced XUV phase shift. Consequently, for a correct interpretation of intensity-dependent dipole phases, it is vital to measure and subtract the B-integral contribution in every solid-state HHG experiment where nonlinear effects are significant. This methodology is broadly applicable to any transmission HHG experiment where the emission phase plays a crucial role, especially when using relatively thick solids, such as the 100-μm samples examined here and thicker materials. Note that after estimating propagation effects such as absorption and dispersion of the generated harmonics in MgO (see the Supplementary Materials), using the complex refractive index data (*53*) and the CXRO database (*54*), we concluded that seeding effects are negligible, confirming the validity of our B-integral calibration approach.

## DISCUSSION

### Theoretical simulations of the harmonic dipole phase

To elucidate the fundamental physical mechanisms governing the dipole phase and aid in analyzing experimental data in MgO, we performed analytical and numerical calculations for two-band and multiband systems (see Materials and Methods). We began with the implementation of an analytical two-band model that examines the semiclassical laser-driven **k**-dependent electron trajectories of the electrons in the CB $\epsilon_{e}^{k}$ and the holes in the VB $\epsilon_{h}^{k}$, akin to those involved in gas-phase HHG. The semiclassical action with electron trajectories that have ionization times $t_{i}$ and recollision times $t_{f}$ was derived from the SBEs under the low electron inversion limit using interband saddle-point approximation (*3*). The dipole phase of the emitted XUV light was then analyzed via the semiclassical action (*20*, *39*)

$$S\left(t_{f}\right)=\int_{t_{i}}^{t_{f}}\Delta \epsilon \left[k\left(\tau \right)\right]d\tau \;\text{with}\;\Delta \epsilon_{k}=\epsilon_{e}^{k}-\epsilon_{h}^{k}$$ (1)

Accordingly, from the semiclassical action, the dipole phase $\phi_{q}$ of harmonic order *q* can be obtained as (*39*)

$$\phi_{q}=q\left(\omega_{0}t_{f}+\frac{\pi }{2}\right)-S\left(t_{f}\right)$$ (2)

**Fig. 3.**
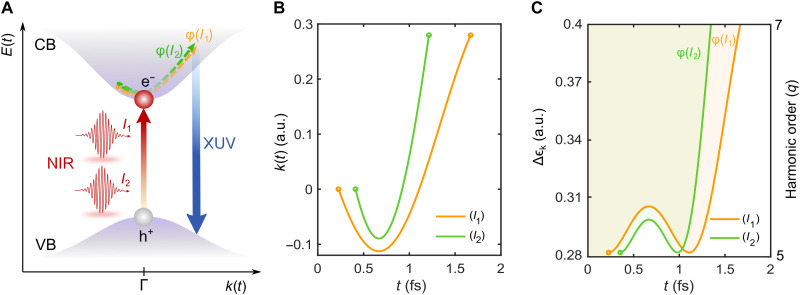
Dipole phase in solid-state HHG. (**A**) Schematic illustration of the intensity-dependent dipole phase trajectories, ϕ(*I*_1_) and ϕ(*I*_2_), induced by the two NIR pulse replicas during HHG in MgO, depicted within the crystal band structure in the reciprocal **k**-space. (**B**) Analytical semiclassical simulations of the dipole phase trajectories for H7 in MgO under applied driving NIR field intensities of 6 TW cm^−2^ (*I*_1_) and 15 TW cm^−2^ (*I*_2_). (**C**) Dipole phases ϕ(*I*_1_) and ϕ(*I*_2_) for H7, obtained using the analytical model shown as time-integrated shaded regions.

While this expression gives the phase contribution to HHG that has its origin in the interband current, which is typically dominant for below-bandgap emission, we show below that the intraband HHG contribution in many cases has a comparable phase profile. Furthermore, the analytical approach makes explicit use of well-defined trajectories, which are often not clearly discernible in more advanced simulations of solid HHG when examining the time-frequency profiles (Gabor transforms) of the HHG emission. As we showed previously by comparing numerical SBE simulations with the analytical model (*55*), the analytical model, despite this shortcoming, predicts the same phases as more advanced numerical simulations that do not show clearly discernible trajectories. The physical interpretation of Eqs. 1 and 2 reveals the origin of the dipole phase in solid-state HHG and is illustrated in Fig. 3A for two intensity-dependent dipole phase trajectories, ϕ(*I*_1_) and ϕ(*I*_2_). In the reciprocal **k**-space, electron-hole pairs are generated because of excitation prompted by the driving NIR field intensities, *I*_1_ and *I*_2_, which are accelerated along separate trajectories (short and long) in the crystal band structure before ultimately recombining in the VB. The accumulated phases of the emitted XUV light for each harmonic order *q* and different trajectories are experimentally measured as the relative dipole phase $\Delta \phi_{q,\text{exp}}$ = ϕ(*I*_1_) − ϕ(*I*_2_), which can also be computed. Figure 3B presents simulation results derived from the two-band analytical model, depicting the dipole phase trajectories as a function of time for H7 and focusing solely on the short trajectories, with *I*_1_ (yellow line) at an intensity of 6 TW cm^−2^ and *I*_2_ (green line) at an intensity of 15 TW cm^−2^. The corresponding dipole phases are calculated using Eqs. 1 and 2, which are shown as shaded areas integrated over time in Fig. 3C. The dipole phase thus maps out the time-integrated motion of the coherently excited electron-hole wave packets, which accelerate through the **k**-space.

Next, the SBEs were employed to numerically simulate the HHG process in MgO solid for a two-band system. The simulations involved using sparse spectral methods (*56*) on a one-dimensional Fourier basis to systematically calculate the microscopic (interband and intraband) contributions to the dipole phase. Comprehensive information regarding the numerical SBE-based simulations can be accessed in our previous publications (*55*, *57*, *58*). The use of Bloch states in two-band calculations, which exhibit complete spatial delocalization, motivates the exploration of an alternative basis comprising states that are tightly localized on specific lattice points, echoing the properties of atomic or molecular orbitals. Considering a realistic material electronic system involving multiple bands (or orbitals), we implemented a real-space, orbital-based framework and performed multiband (full electronic band structure) numerical simulations of the dipole phase in MgO using a basis derived from maximally localized Wannier orbitals (*59*, *60*). Note that all theoretical simulations presented in this study were carried out for MgO solid along the Γ-X high-symmetry crystal direction, which pertains to the Mg─O bond. The experiments were performed in this orientation, as confirmed by the polarization orientation scans in MgO (see fig. S6). Additional simulations along the Γ-K-X direction (or Mg─Mg bond) have also been conducted and can be found in the Supplementary Materials.

**Fig. 4.**
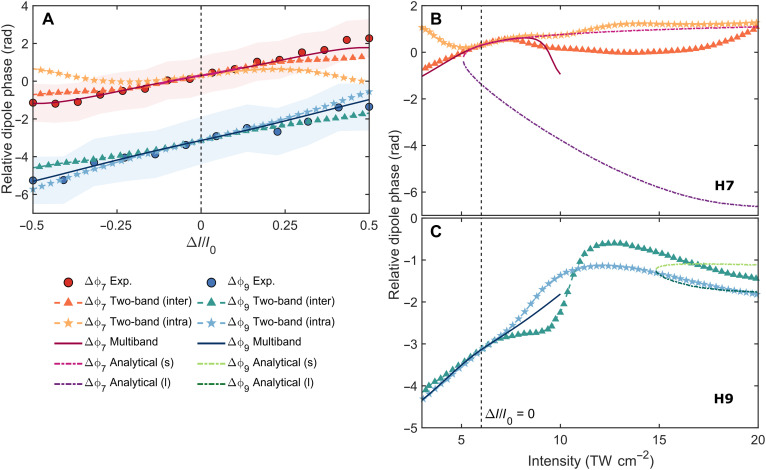
Intensity-induced relative dipole phase for $\Delta \phi_{7}$ and $\Delta \phi_{9}$ in MgO with the 800-nm driver. (**A**) Experimental $\Delta \phi_{7}$ (red dots) and $\Delta \phi_{9}$ (blue dots) results for MgO along the Γ-X (Mg─O bond) direction in comparison with the analytical semiclassical and numerical two-band simulations, as well as multiband simulations, determined within the measured intensity range of Δ*I*/*I*_0_ = ±0.5. The reference point at Δ*I*/*I*_0_ = 0 (*I*_0_ = 12 TW cm^−2^) corresponds to equal intensities of 6 TW cm^−2^ in the NIR foci (vertical dashed black line). The error bars (shaded areas) are 2σ uncertainties derived from the linear fits of the experimental fringe patterns. (**B** and **C**) Simulated $\Delta \phi_{7}$ (H7) and $\Delta \phi_{9}$ (H9) dipole phase results, shown in (A), presented on an absolute intensity scale and extended to higher driving laser peak intensities ranging from 3 to 20 TW cm^**−2**^, encompassing all computational findings. Note that the analytical calculations consider only 1 OC of the 800-nm laser field, whereas the numerical two-band analysis accounts for 8 OC. For the best comparison with experimental results, the theoretical curves are vertically offset, with their reference point aligned at 6 TW cm^−2^.

Figure 4 shows the experimental and calculated relative dipole phase as a function of the driving laser peak intensity for the H7 and H9 of the fundamental 800-nm field in MgO. For both $\Delta \phi_{7}$ and $\Delta \phi_{9}$, the analytical semiclassical calculations are presented for the short (s) and long (l) electron trajectories, while the numerical simulations based on the SBE two-band and Wannier state multiband models show no behavior indicative of exclusively short or long trajectories (*55*). Figure 4A displays a comparison between the experimental dipole phase results for $\Delta \phi_{7}$ (red dots) and $\Delta \phi_{9}$ (blue dots) and three theoretical models: the analytical (s) model (pink dashed-dotted line), the numerical two-band model for interband (inter) transitions (orange triangles) and intraband (intra) transitions (yellow stars), and the multiband model (ruby solid line) for H7. For H9, the figure includes the numerical simulation results obtained from the two-band model for the intraband (light blue stars) and interband (blue-green triangles) transitions and the multiband model (navy blue solid line). For a direct comparison with the experimental data, the initial theoretical dipole phase results (Fig. 4, B and C), calculated on an absolute intensity scale, are presented for the Δ*I*/*I*_0_ = ±0.5 range in Fig. 4A. The reference point is at Δ*I*/*I*_0_ = 0 (*I*_0_ = 12 TW cm^−2^), meaning 6 TW cm^−2^ in each NIR focal spot (indicated by the vertical dashed black line). The experimental results for $\Delta \phi_{7}$ and $\Delta \phi_{9}$ in Fig. 4A correspond to the data points shown in Fig. 2 (A and B), incorporating the harmonic-weighted nonlinear phase contributions (B-integral) induced at 800 nm. The error bars, depicted as red (H7) and blue (H9) shaded areas in Fig. 4A, were defined from linear fits of the XUV and 800-nm fringe patterns. Table 1 summarizes the relative dipole phase values for H7 and H9 in MgO as obtained from experimental data and multiband and two-band numerical models. The results clearly show that the theoretical dipole phase calculations for H7 in the low-intensity regime (Δ*I*/*I*_0_ = ±0.3), corresponding to *I*_1_ and *I*_2_ variations from 4.2 to 7.8 TW cm^−2^ in each arm (inversely), are in good agreement with one another and with the experimental $\Delta \phi_{7}$ data, reflecting the largely semiclassical nature of short electron trajectories. At higher intensities (Δ*I*/*I*_0_ = ≳0.5), the multiband effects likely become significant. Eventually, considering effects such as carrier-carrier and carrier-phonon scattering will become important, which are currently not included in our model. All named effects are especially important as the real-space trajectory length increases. This inherently suggests that the atomic dipole phase concept used in gas-phase HHG is less applicable to solids under higher driving intensities. This is corroborated by the observation that the electronic band structure of MgO around the Γ-point remains approximately parabolic at low driving intensities (fig. S17), indicative of free particle–like behavior in a low driving intensity setting. As the driving intensity increases, the effective momenta rise, enabling carriers to access energy levels that are offset from those of a free particle. In the case of H9, the experimental $\Delta \phi_{9}$ behavior is almost flawlessly replicated by the numerical two-band and multiband models. Conversely, the semiclassical approach fails to align with the experimental data because of the emission threshold of H7 being 5 TW cm^−2^ compared to H9’s higher requirement of around 15 TW cm^−2^, as depicted in Fig. 4C. From a classical perspective, this can be attributed to the relationship between photon energy (*E*_ph_ *= E*_g_ *+ E*_kin_) and laser intensity, where *E*_kin_ ∝ *k*^2^(*t*) increases with intensity.

**Table 1.** Comparison of experimental ($\Delta \phi_{q}$) and calculated ($\Delta \phi_{q,\text{calc}}$) relative dipole phase values for the H7 and H9 of the 800nm driving field in MgO along the Γ-X crystal direction (Mg─O bond) across a varied Δ*I*/*I*_0_ = ±0.5 range (*I*_0_ ~ 12 TW cm^−2^).

Harmonic order (*q*)	$\Delta \phi_{q}$ (rad)	$\Delta \phi_{q,\text{calc}}$* (rad)	$\Delta \phi_{q,\text{calc}}$† (rad)	$\Delta \phi_{q,\text{calc}}$‡ (rad)
7	3.52 ± 1.01	2.96	2.04	0.74
9	3.90 ± 1.26	4.34	2.93	5.16

* Multi-band numerical SBE model.

† Two-band numerical SBE model (interband).

‡ Two-band numerical SBE model (intraband).

**Fig. 5.**
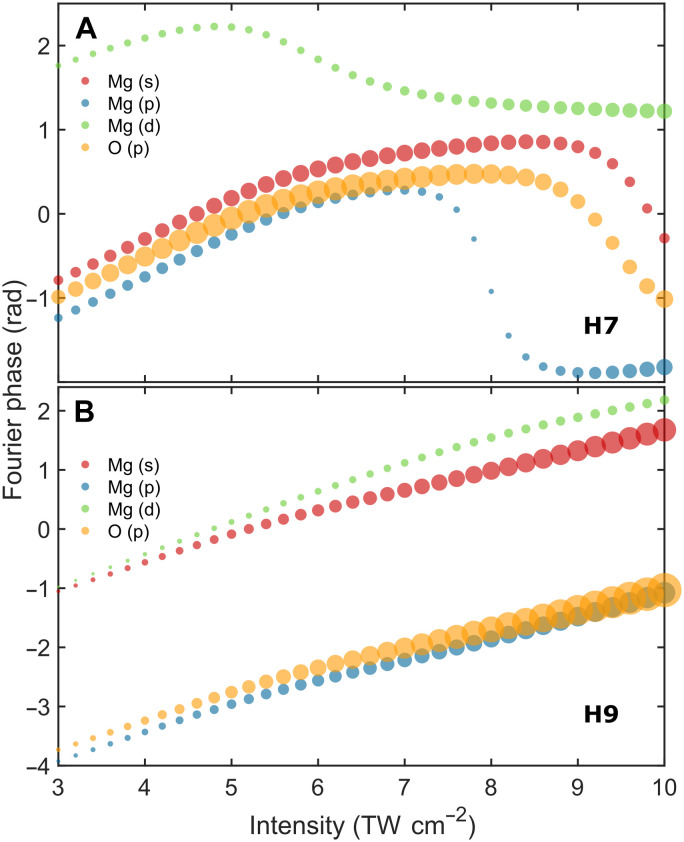
Orbital contributions to the harmonic relative dipole phase in MgO. Fourier phases, weighted by the amplitudes of the three Mg orbitals (s, p, and d) and one O orbital (p) as a function of the driving laser peak intensity for H7 (**A**) and H9 (**B**) in MgO, based on the numerical multiband simulations. The size of each circle corresponds to the Fourier amplitude associated with the individual orbital, while color coding is used to distinguish between them visually. The results are displayed for the linearly polarized 800-nm driving laser pulses aligned along the Mg─O bond in the Γ-X crystal direction of MgO.

In Fig. 4 (B and C), all computed results are shown on an absolute intensity scale for H7 and H9, respectively, extended to driving laser peak intensities up to 20 TW cm^−2^. Figure 4B displays additional findings for $\Delta \phi_{7}$ simulated for long electron trajectories through the analytical model (purple dashed-dotted line). Furthermore, in Fig. 4C, we present the results of the semiclassical calculations for both short (light green dashed-dotted line) and long (dark green dashed-dotted line) trajectories for $\Delta \phi_{9}$. The analytical model demonstrates that the dipole phase slope changes more rapidly for long trajectories than for short ones, a phenomenon attributed to the longer electronic quantum path present in gas-phase HHG, as reported in earlier studies (*14*, *39*). Notably, the analytical model of the short trajectories agrees well with both experiment and other theories, while the long-trajectory analytical model is off. As explained above and in (*55*), solid-state HHG usually does not show any signatures of long-trajectory contributions, which is thus confirmed by the phase measurements. We also note from the numerical calculations that the interband and intraband phase contributions differ for H7 at low intensities (<5 TW cm^−2^) and are substantially similar in the intermediate experimentally accessible intensity range (from 5.8 to 7.3 TW cm^−2^). This measurement thus confirms the interband current as the origin for H7, in accordance with previous literature findings (*61*). The similar phase accumulation for interband and intraband current likely has its origin in the few-band nature of HHG from MgO in the observed range. While interband harmonics are inherently sensitive to interband phases and, thus, to energy differences between bands, the intraband harmonics will be sensitive to intraband energy differences as a function of carrier momentum, which will result in a similar phase if the VB and CB are both contributing with a similar emission amplitude and band curvature. The dominant contribution of the VB and CB to H7 and H9 in the measured intensity range is further corroborated by decomposing the phase contributions into their molecular orbital origins in Fig. 5.

### Orbital-resolved contributions to the harmonic dipole phase

We delved deeper into the physical origins underlying the dipole phase by assessing the various orbital contributions to the harmonic dipole phase in MgO using an orbital-based framework on the Wannier basis. Figure 5 illustrates the contributions of three magnesium (Mg) orbitals (s, p, and d) and one oxygen (O) orbital (p) to the relative dipole phase of harmonics H7 (Fig. 5A) and H9 (Fig. 5B), as determined by Fourier phases and amplitudes calculated over an intensity range of 3 to 10 TW cm^−2^ through multiband numerical analysis. The results reveal several key findings: For H7, the dipole phase at lower driving intensities (<8 TW cm^−2^) is mainly affected by nearly equal contributions from the Mg (s) and O (p) orbitals. However, at higher applied intensities, the respective contributions of the Mg (s) and O (p) orbitals decrease, and the contributions of the Mg (p) and Mg (d) orbitals become prevalent. For H9, the phases of all orbitals increase linearly with intensity, with the O (p) orbital making the largest contribution, thereby primarily determining the overall harmonic dipole phase. The dominant contributions of Mg (s) and O (p) orbitals in MgO are expected, as they have been identified as the primary contributors to the material’s electronic properties in the literature (*62*). As evident by the projected density of states simulations (fig. S17), the highest occupied molecular orbital (HOMO) is dominated by the O (p) orbitals, whereas the lowest unoccupied molecular orbital (LUMO) is dominated by Mg (s). This coincides with the intuitive ionic bonding view, where the more electronegative O atom attracts two electrons from Mg. Therefore, the measured phases at low and moderate intensities of both H7 and H9 are dominated by electron dynamics in the HOMO and LUMO of MgO. At higher intensities, the contributions from orbitals other than HOMO and LUMO become dominant as the electrons can be promoted into those orbitals too. This orbital picture on the origin of harmonic phases is equivalent to the more common band-structure view of solid-state HHG, where excitation prepares coherent electron-hole pairs, and laser-driven electron dynamics further promote these excited carriers into higher- and lower-lying bands. Complementary simulations for harmonic 5 (H5, 8 eV) in MgO, not observed experimentally in this study but computed (see fig. S18), further reinforce the notion that Mg (s) and O (p) orbitals significantly contribute to the dipole phase, including within the bandgap regime.

### Conclusions and outlook

This study has provided a detailed examination of the intensity-dependent dipole phase in solid-state HHG through the use of ultrastable XUV interferometry. The synthesis of experimental results with multifaceted theoretical insights into the harmonic phase in MgO has yielded the following key findings: The generation of high harmonics using the 800-nm driver in the above-bandgap regime suggests that the interband mechanism plays a key role in the emitted phase. Moreover, the insights gained from this work not only deepen our understanding of the fundamental processes involved in HHG from solids but also pave the way for better control over macroscopic phase matching and the divergence and focusing characteristics of the emitted XUV light. This is particularly enabled by establishing and experimentally verifying an analytical model for the dipole phase, which can serve as input for simulations of phase matching and HHG wavefronts.

By carefully manipulating the driving laser intensity, one can achieve attosecond-level tuning of the harmonic phase in solid-state HHG. This could have profound applications in fields such as attosecond science, quantum optics, and high-resolution imaging. In addition, our approach not only facilitates the direct measurement of the harmonic dipole phase but also encapsulates sensitivity to the B-integral, thereby providing a robust framework for exploring nonlinear effects in real time. Future research should aim to explore how these nonlinear effects influence HHG in a range of materials with distinct optical properties, including nonlinear crystals, amorphous solids, and nanostructures, as this could facilitate the development of compact, efficient, and stable all-solid-state XUV light sources, providing high-brightness light for probing intricate material properties at the nanoscale.

## MATERIALS AND METHODS

### Experimental details

A Ti:sapphire laser amplifier system (Astrella, Coherent) was used to produce NIR pulses with an 800-nm central wavelength (1.55-eV photon energy), 7-mJ pulse energies, and 45-fs pulse durations at a 1-kHz repetition rate. To generate the ultrashort NIR pulse replicas, 4-mJ pulse energies were driven to a birefringent common-path interferometer assembled from two pairs of alpha-barium borate wedges [see details in the Supplementary Materials (fig. S1)]. The resulting two pulse replicas were directed to an *f* = 500 mm silver spherical mirror, which focused the beams into a solid target to generate the XUV pulses through the HHG process. The double-side polished, 100-μm-thick, (100)-cut bulk crystalline MgO sample (purchased from SurfaceNet GmbH) was chosen as a solid HHG target. In the experiments, the linearly polarized driving laser pulses (s-polarized) were aligned with their polarization direction fixed along the Γ-X direction (Mg─O bond) of the solid. Focusing the NIR pulse replicas, derived from attenuated ~20-μJ pulse driving laser energy into the sample, led to a total spatiotemporal peak intensity of ~12 TW cm^−2^, with 6 TW cm^−2^ at each focus. The two foci of NIR pulse replicas with a spot size of 50 ± 0.46 μm (full width at half maximum) were spatially separated vertically on the MgO target by a distance ~1.6 times the beam spot diameter (162 ± 0.15 μm) (fig. S2). After generating two XUV pulses from both foci, they were spatially overlapped in the far field in a vacuum detection chamber. An aberration-corrected, concave, flat-field, diffraction grating (Shimadzu, 1200 grooves/mm), housed in the detection chamber, was used to spectrally disperse the harmonics onto a double-stack multichannel plate) and a phosphor screen detector (Photonis USA). The back of the phosphor screen was imaged by a complementary metal-oxide semiconductor camera (Basler ace, acA1300-200 μm), mounted outside the detection chamber. For HHG and XUV interferometry, we used a low-vibration turbomolecular pump (Edwards, STP-XA3203C), which was backed by a scroll pump (Pfeiffer, SEK 28/40), maintaining chamber pressures below 10^−8^ mbar during the experiments.

### Numerical and analytical models

We simulate the dipole phase in MgO along a single high-symmetry crystal direction, Γ-X (Mg─O bond) or Γ-K-X (Mg─Mg bond), using either two-band or multiband (full band structure) models in conjunction with SBEs. In the numerical and analytical two-band models, we use the electronic band structure of cubic MgO (100-cut) (*63*) for a two-level system that includes the first VB and first CB. The computations are performed for the MgO crystal structure, which has a bandgap $E_{g}$ = 7.8 eV (*64*), a lattice constant *a* = 4.19 Å, a VB height of 1.1 eV, a CB height of 5.5 eV, a transition dipole moment at the Γ-point $d_{0}$ = 0.78 a.u. (atomic units), and a dephasing time $T_{2}$ = 3 fs. In the numerical two-band model, one high-symmetry crystal direction of MgO (either Γ-X or Γ-K-X) is represented by a one-dimensional chain, with the assumption of tight-binding sinusoidal bands. The system is driven well below resonance with 800-nm Gaussian-envelope laser pulses comprising 8 OC, with τ_800_ = 2.66 fs for 1 OC. The specifics of our numerical model are detailed in prior publications (*55*, *57*, *58*).

The analytical semiclassical model is derived from the laser-driven electron trajectories. This is achieved using the SBE by applying saddle-point approximation to the interband current while assuming low carrier inversion (*3*). The trajectories are computed for 1 OC of the driving 800-nm laser field. Note that the intraband current is completely neglected in this model.

To obtain the trajectories, we consider the time-dependent effective carrier momentum during acceleration, which follows that of the driving electric field E(t) as

$$k\left(t\right)=A\left(t\right)-A\left(t_{i}\right)+k_{0}$$ (3)

where $t_{i}$ is the excitation time, A(t) is the vector potential, and $k_{0}$ the initial momentum of the electron (hole). We consider a cosine laser field, with its vector potential defined as

$$A\left(t\right)=-\int E\left(t\right)\mathrm{dt}=-\frac{E_{0}}{\omega_{0}}\text{sin}\left(\omega_{0}t\right)$$ (4)

with peak electric field $E_{0}$ and frequency of the laser field $\omega_{0}$. Here, we neglect tunneling and restrict excitation to the Γ-point ($k_{0}=0$), resulting in distinct long and short trajectories, similar to those observed in gases (*65*). Note that these distinct trajectories are not present in the numerical SBE simulations, as they facilitate excitation across the entire k-space of the bands.

The real-space trajectories per band λ∈{e,h} for the different $t_{i}$ values are obtained by integration of the carrier group velocity $v_{\lambda }=\delta_{k}\epsilon_{\lambda }^{k}$ over time, resulting in

$$x_{\lambda }\left(t\right)=\int_{t_{i}}^{t}v_{\lambda }\left[k\left(\tau \right)\right]d\tau$$ (5)

The group velocity is derived from the band structure of MgO, adopting the same two-band configuration as in the numerical SBE simulations.

As the real-space distance between the electron and hole reaches zero, recombination with the time $t_{f}$ occurs, establishing the condition

$$\Delta x\left(t_{f}\right)=x_{e}\left(t_{f}\right)-x_{h}\left(t_{f}\right)=0$$ (6)

Only the trajectories that undergo recombination contribute to the interband current, with the corresponding photon energy being the energy difference between the charge carriers at the time of their recombination. Thus, the analytical semiclassical model is limited to evaluating harmonics that have energies greater than the bandgap but less than the maximum energy difference between the CB and the VB.

As discussed earlier in the main text, the phase of the emitted XUV light for each trajectory can be represented by the semiclassical action (*20*)

$$S\left(t_{f}\right)=\int_{t_{i}}^{t_{f}}\Delta \epsilon \left[k\left(\tau \right)\right]d\tau \;\text{with}\;\Delta \epsilon_{k}=\epsilon_{e}^{k}-\epsilon_{h}^{k}$$ (7)

Therefore, the dipole phase for harmonic order *q* can be derived from the semiclassical action as follows (*39*)

$$\phi_{q}=q\left(\omega_{0}t_{f}+\frac{\pi }{2}\right)-S\left(t_{f}\right)$$ (8)

In the numerical multiband model, we first calculate the field-free band structure and dipole couplings for MgO through density functional theory using the electronic band structure code Quantum Espresso (*66*). The electronic structures and dipolar matrix elements were calculated using the Heyd-Scuseria-Ernzerhof hybrid functional, which produces a bandgap within ~15% of the experimental value and about 7% of the semiempirical Becke-Johnson exchange potential (*67*). We use the Heyd-Scuseria-Ernzerhof exchange-correlation hybrid functional on a Monkhorst-Pack grid of 10 by 10 by 10 points. The Bloch states are projected onto a set of maximally localized Wannier functions using the s, p, and d orbitals of Mg and the p orbitals of O with the code Wannier90 (*68*). To ensure an accurate representation of the bandgap, a rigid scissor shift was subsequently applied to match the experimental bandgap of 7.8 eV (*69*). The calculated band dispersion and the orbital-resolved projected density of states are presented in fig. S17. The time-dependent propagation is subsequently performed using the density matrix formalism with the code detailed in (*59*), incorporating the experimental laser parameters (intensity, frequency, and pulse duration). Decoherence effects are included phenomenologically via a dephasing time $T_{2}$ = 3 fs. We observe only small shifts to the dipole phase when changing $T_{2}$ from 3 to 6 fs in the harmonic and intensity range of interest. The orbital contributions to the current are extracted following the procedure in (*60*).

## Supplementary Materials

### This PDF file includes:

Notes S1 to S3 Figs. S1 to S24
