# Supplementary material for: Attosecond high-harmonic interferometry probes orbital- and band-dependent dipole phase in magnesium oxide
Source: Sci Adv. 2026 May 1;12(18):eaeb4109. doi: 10.1126/sciadv.aeb4109 (PMC13134606; doi:10.1126/sciadv.aeb4109)
Supplement: Supplementary file 1 — Notes S1 to S3 Figs. S1 to S24 [file sciadv.aeb4109_sm.pdf]

Science Advances
AAAS

Supplementary Materials for

Attosecond high-harmonic interferometry probes orbital- and band-dependent dipole phase in magnesium oxide

Nataliia Kuzkova *et al.*

Corresponding author: Nataliia Kuzkova, n.kuzkova@arcnl.nl; Peter M. Kraus, p.kraus@arcnl.nl

Sci. Adv. **12**, eaeb4109 (2026)
DOI: 10.1126/sciadv.aeb4109

This PDF file includes:

Notes S1 to S3
Figs. S1 to S24

Supplementary Text

Note S1. Experimental details

S1.1. XUV interferometric setup

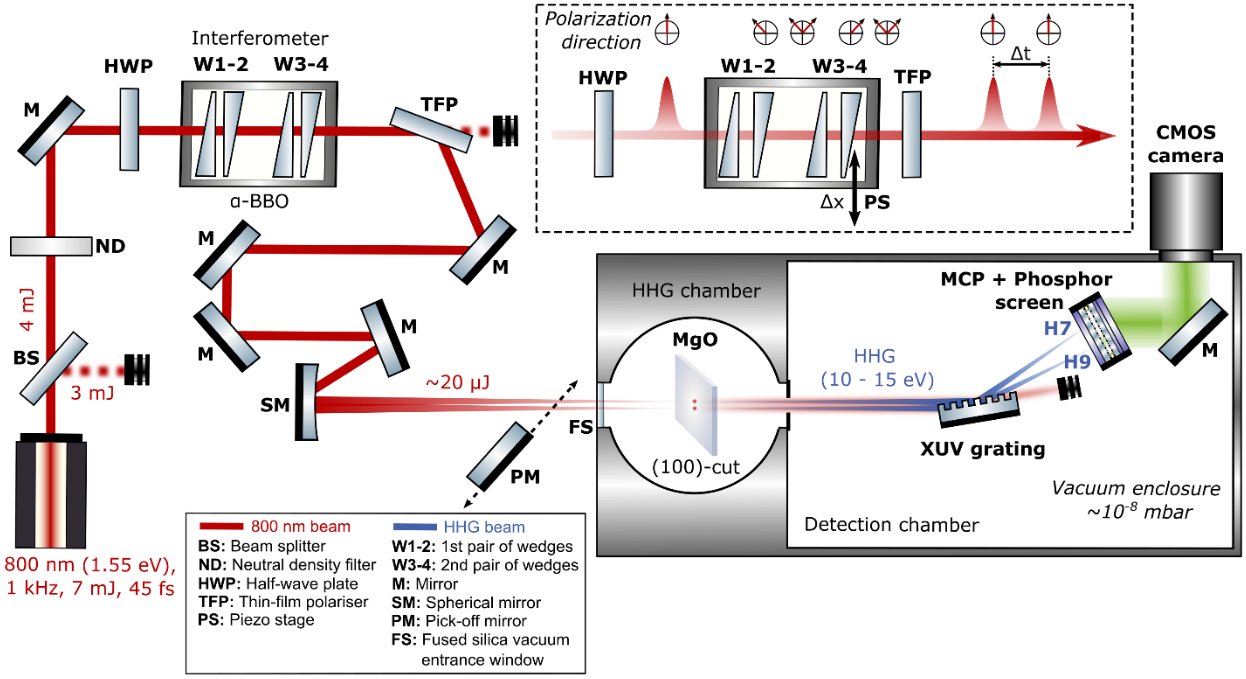

Fig. S1. Schematic overview of the main components of the XUV interferometric setup.

Fig. S1 illustrates a schematic overview of the main components of the XUV interferometric setup. The Materials and Methods section of the main text provides a comprehensive description of the laser system and its key components. Here, we focus on the details of a birefringent common-path interferometer, which generated the ultrashort near-infrared (NIR) pulse replicas. The propagation of the fundamental NIR beam through and after the interferometer, including polarization direction diagrams (red arrows), is illustrated in the inset of Fig. S1. The interferometer consists of two pairs of 20x20x1 mm alpha-barium borate (α-BBO) birefringent wedges (United Crystals Inc.), with an optical axis 45° away from 20 mm edges and an apex angle, $\alpha = 14^{\circ}$, which corresponds to a relatively large birefringence, $\Delta n = 0.1189$ at 800 nm wavelength, where Δn is the difference between the refractive indices of extraordinary, n_e, and ordinary, n_o, polarization, respectively. Orientation of the optical axis of the first pair of wedges (W1-2) at an angle of 45° with respect to the input linearly polarized NIR beam (vertical s-polarization with respect to the laser table) resulted in the generation of two orthogonally polarized phase-locked collinear replicas along the propagation direction. These two pulse replicas with the same intensity distribution were then sent through a second pair of wedges (W3-4) whose optical axis was perpendicular to W1-2. As a result, the relative delay, $\Delta\tau$, between the two orthogonally polarized pulse replicas was introduced by the interferometer, which can be expressed as [46]:

$$\Delta\tau = \frac{\Delta n \cdot \Delta x}{c} tan\alpha, \tag{S1}$$

where Δx represents the displacement of one of the wedges achieved by shifting it transversely relative to the laser beam and changing its thickness, and c is the speed of light in vacuum.

By displacing the last wedge (W4), which was mounted on a linear piezo stage (SmarAct, SLC-1740s, 26 mm travel range, 1 nm resolution), the sub-20-attosecond $\Delta\tau$ interferometric temporal precision was achieved. The two orthogonally polarized NIR pulse pairs produced after the interferometer were subsequently aligned to the same linear polarization axis (s-polarization) by utilizing a broadband thin film polarizer (TFP) purchased from Altechna. An ultrathin half-wave plate (HWP, Newlight Photonics Inc.) placed before the interferometer was used to control the orientation of the input laser polarization axis.

S1.2. Characterization of the NIR foci

The size and separation distance between the two NIR foci on the MgO sample were defined from separate measurements performed in air using a beam profiling camera (Gentec-EO, BEAMAGE-4M, 5.5 µm pixel size). To achieve this, a pickoff silver mirror (PM) was positioned before the vacuum HHG chamber to reflect the two attenuated fundamental laser beams onto the camera (Fig. S1). By slightly tilting the first wedge (W1), the focal spots of the NIR beams were fine-tuned, thus controlling the vertical separation distance between them. Fig. S2(**A**) shows the 2D spatial intensity profiles of the 800 nm beams measured at the focal plane. The corresponding vertical (y-axis) intensity profiles of the NIR foci, extracted at their peak maxima, considering the minimum pixel size of the profiler, were fitted using a superposition of Gaussian envelopes and are presented in Fig. S2(**B**). The focal spot size for each NIR beam was found to be $\omega_0 = 50 \pm 0.46$ µm (full-width at half-maximum, FWHM). The two-foci separation distance was determined to be 162 ± 0.15 µm, which is nearly 1.6 times the beam spot diameter. The small peak located between the foci in Fig. S2(**B**) is due to an artifact from the neutral density filters applied to reduce peak intensities on the beam profile camera during data acquisition.

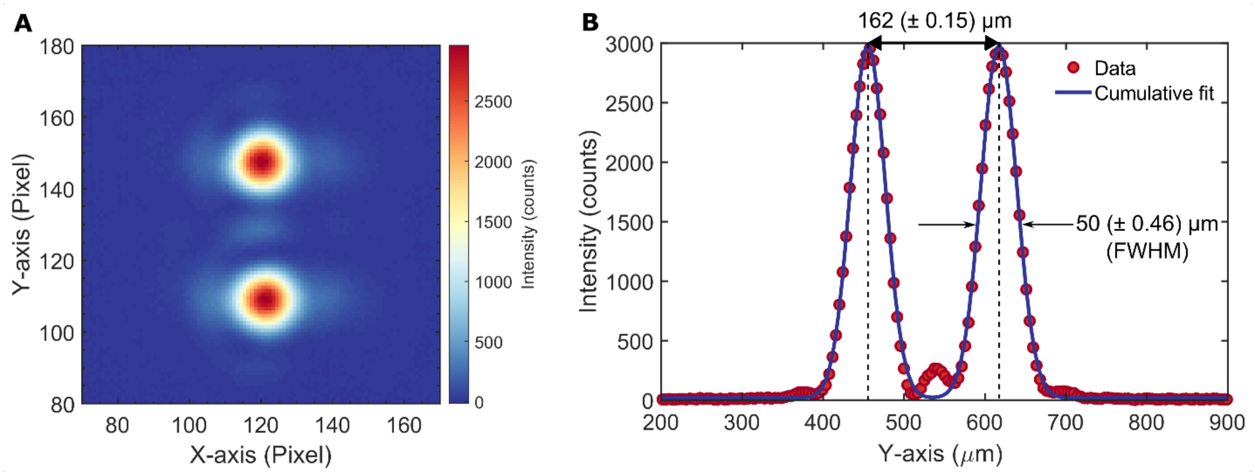

Fig. S2. Spatial intensity profiles of the NIR foci.

(**A**) The image of 2D spatial intensity profiles of the two NIR foci at $\frac{\Delta I}{I_0} = 0$. (**B**) The vertical intensity profiles of the NIR foci (red dots), extracted at their peak maxima from panel (**A**) fitted to a superposition of Gaussian envelopes. The blue line represents the cumulative fit result, resulting in each beam focal spot size of $\omega_0 = 50$ µm (FWHM). The peak positions indicated by the dashed lines were used to determine the separation distance of 162 µm between the two foci.

In our time-dependent XUV studies, we observed that the influence of the fundamental frequency component on the harmonic phase becomes substantial when the distance between the NIR focal spots is <1.5 times the beam spot diameter. To prevent 800 nm frequency oscillations and guarantee that harmonic signals originate from independent foci, we maintained a separation of ≥1.5 times the beam spot diameter throughout all experiments.

S1.3. Intensity calibration of the NIR pulses

The total energy of the driving NIR pulses, E_p, was measured using a broadband thermal power sensor (Thorlabs, S401C) positioned just before the vacuum-fused silica entrance window, prior to the MgO sample, resulting in ~20 µJ per pulse. The pulse duration, τ_p, was characterized after the interferometer and before the focusing spherical mirror (SM) using a home-built second harmonic generation frequency resolved optical gating (SHG-FROG) apparatus, yielding a duration of τ_p = 47 ± 0.6 fs (FWHM), as shown in Fig. S3.

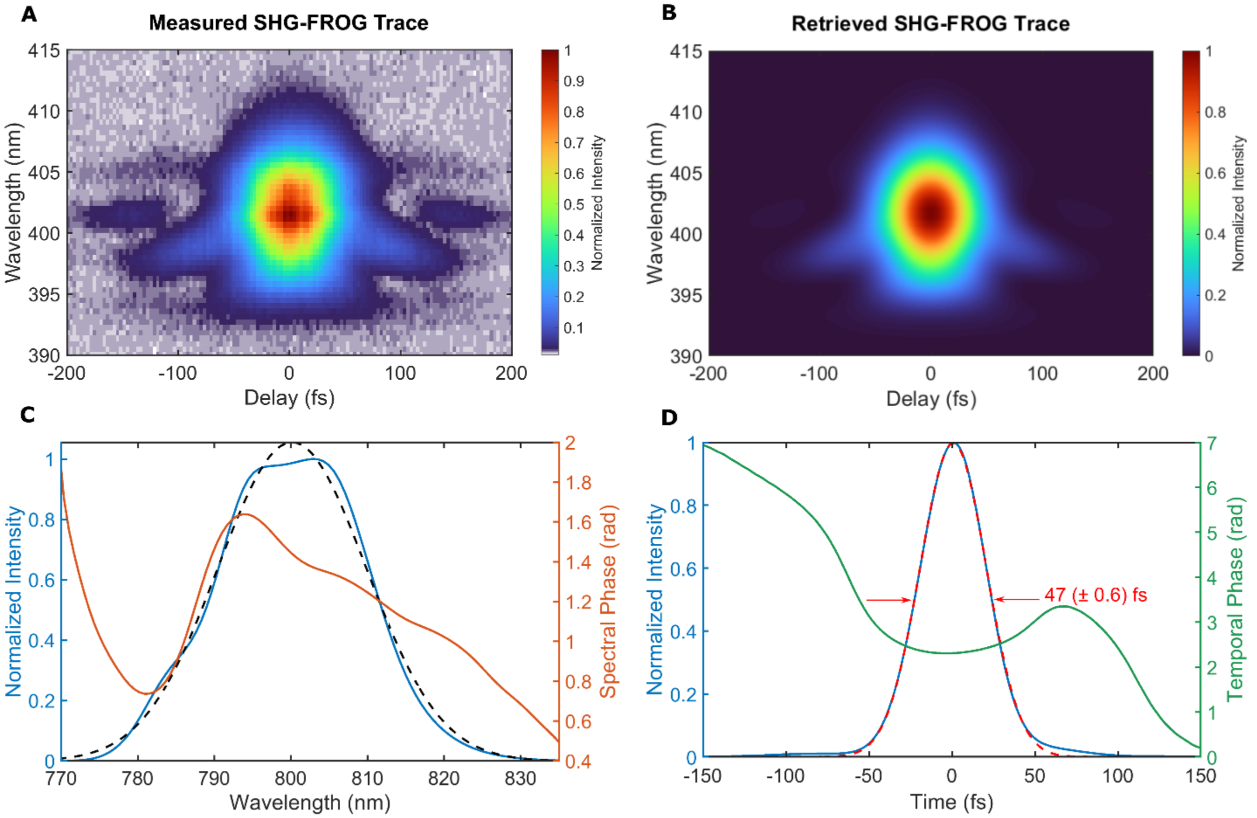

Fig. S3. Characterization of the NIR laser pulse duration by SHG-FROG.

The measured (**A**) and retrieved (**B**) SHG-FROG traces of the NIR laser pulses after the interferometer, prior to focusing. (**C**) The reconstructed spectrum (dashed black line), spectral phase (orange line), and independently measured spectrum (blue line). (**D**) The reconstructed pulse envelope (blue line) with corresponding temporal phase (green line). The red dashed line indicates a Gaussian fit to the reconstructed pulse envelope, with red arrows marking the pulse duration of 47 ± 0.6 fs (FWHM) derived from the fit.

Assuming the Gaussian spatial and temporal profiles, the incident peak intensity in vacuum, I_{vac}, of the driving NIR field was defined as:

$$I_{vac} = 1.88 \cdot \frac{E_p}{\pi\left(\omega_{1/e^2}\right)^2 \tau_p}, \quad \text{(S2)}$$

Here, ω_{1/e^2} is the focal spot diameter at the 1/e^2 intensity level, defined as $\omega_{1/e^2} = \frac{\omega_0}{\sqrt{2\log(2)}}$, were ω_0 is the measured beam focal spot size at FWHM. The factor of 1.88 arises from intergation of the Gaussian spatial and temporal profiles, ensuring that the calculated intensity reflects the peak intensity at the center of the beam, given the pulse energy and beam waist size.

The inside-sample total peak intensity, I_0, was derived from the incident vacuum intensity, I_{vac}, by accounting for surface reflections via Fresnel equations. This calculation incorporated the dielectric function of a non-absorbing medium at normal incidence and the effect of the refractive index, n, on energy flux, expressed as:

$$I_0 = I_{vac} \cdot \frac{4n_{MgO}}{\left(1+n_{MgO}\right)^2} \cdot \left(\frac{4n_{FS}}{(1+n_{FS})^2}\right)^2 \approx I_{vac} \cdot 0.93 \cdot 0.96^2, \quad \text{(S3)}$$

where n_{MgO}=1.7276 and n_{FS}=1.4533 represent the indices of refraction of the MgO sample, and the fused silica window, respectively, at the 800 nm. The third term accounts for the reflection of two NIR pulses from the vacuum-fused silica (FS) entrance window (see Fig.S1).

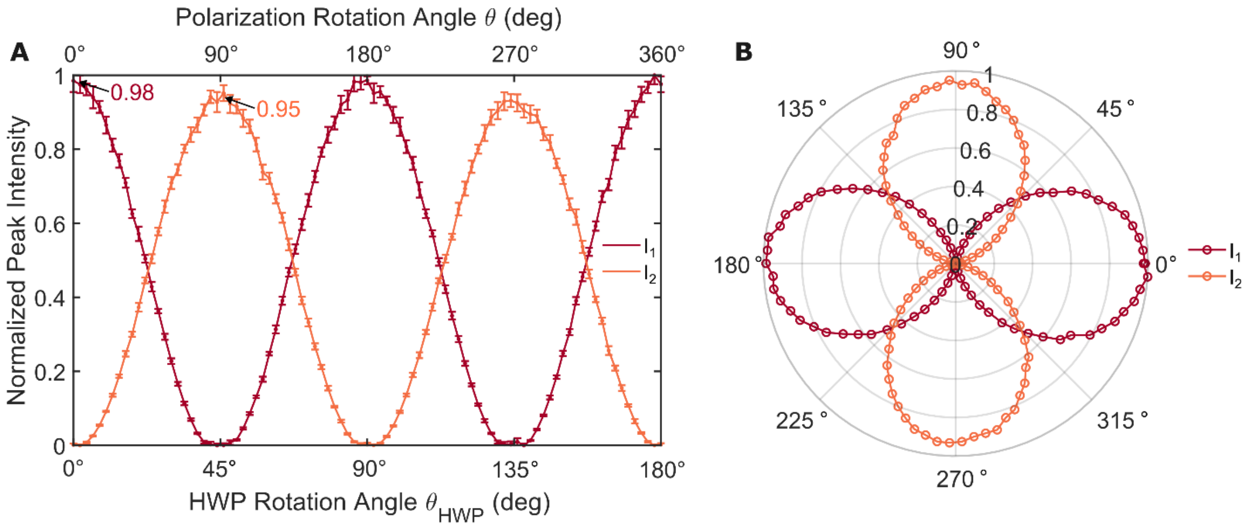

Fig. S4. Variation of the NIR foci peak intensities with the HWP rotation angle.
(**A**) Normalized peak intensities of the NIR foci, I_1 (red) and I_2 (orange) as a function of the HWP rotation angle θ_{HWP} (bottom x-axis) and the laser polarization angle θ (top x-axis). The peak maxima: I_1 at $\theta = 0°$ of 0.98 and I_2 at $\theta = 90°$ of 0.95, depicted in the figure, indicate a difference of 3 ± 0.03% at the $\frac{\Delta I}{I_0} = \pm 1$ positions during the interferometric experimets. The error bars denote the $\pm 2\sigma$ uncertainty. (**B**) Polar plot of data presented in panel (**A**) as a function of θ.

In the intensity-dependent XUV and 800 nm interferometric measurements, the relative peak intensities of the NIR foci, I_1 and I_2, were varied by rotating the HWP positioned prior to the

interferometer (Fig. S1) by an angle θ_{HWP} from 0° to 45°, with an increment of 1°. The position of $\theta_{HWP} = 0°$ corresponded to the $\frac{\Delta I}{I_0} = -1$, whereas at $\frac{\Delta I}{I_0} = 1$, $\theta_{HWP} = 45°$. Before conducting the experiments, we examined wheather the θ_{HWP} angle tuning exhabited a linear relationship with the intensities I_1 and I_2, i.e., whether the peak intensities of both NIR foci matched at the $\frac{\Delta I}{I_0} = \pm 1$ positions. Consequently, a full scan of the HWP angle θ_{HWP} was performed from 0° to 180°, equating to a laser polarization rotation θ from 0° to 360° ($\theta = 2\theta_{HWP}$).

Fig. S4(**A**) shows the normalized peak intensities of the NIR beams measured as a function of the HWP rotation angle θ_{HWP} (bottom x-axis) and the laser polarization angle θ (top x-axis). The same data is represented in a polar plot in panel (**B**), as a function of the laser polarization angle. As can be seen from the figure, a slight difference of 3 ± 0.03% was observed between the peak intensities of beam 1 (I_1) at θ = 0° and beam 2 (I_2) at θ = 90°, where all intensity is concentated into a single beam. This implies that the interferometric measurements were not performed precisely at $\frac{\Delta I}{I_0} = 0$, a correction for which is detailed below.

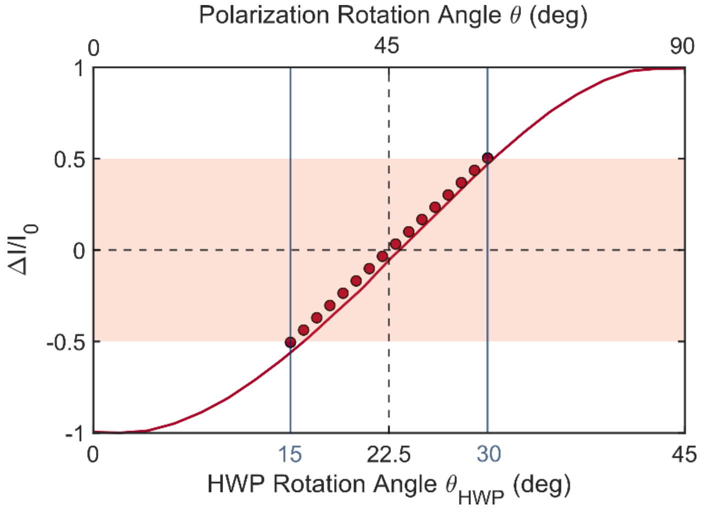

Fig. S5. Correlation between the $\frac{\Delta I}{I_0}$ and HWP rotation angle.

The relationship between the NIR foci peak intensities variation $\frac{\Delta I}{I_0} = \pm 1$ and the HWP rotation angle θ_{HWP} (0°–45°) comparing the experimentally measured XUV interference fringe shift maxima of H7 in MgO (red dots) with the simulated intensity trend (red line). The experimental intensity range of $\frac{\Delta I}{I_0} = \pm 0.5$ is indicated by the orange shaded area, corresponding to HWP angles between 15° and 30° (blue vertical lines).

The intensity signals of the interference fringes presented in Fig. 2 of the main text show minor discrepancies on the positive and negative sides of x-axis ($\Delta I/I_0$), which are caused by imperfections in the HWP (Fig. S4) and the subsequent calibration of the zero position on the intensity scale to offset these effects. Fig. S5 illustrates an evaluation of the linear relationship between NIR beam intensities and the HWP rotation angle θ_{HWP} spanning from 0° to 45°, corresponding to the $\frac{\Delta I}{I_0} = \pm 1$ range. The figure compares the experimental relative fringe shift XUV data, $\Delta\varphi_{7,exp}$, derived from the local fringe maxima at the H7 peak in MgO (red dots), with

the simulated intensity curve (red line). From the figure, it can be observed that, within the experimental $\frac{\Delta I}{I_0} = \pm 0.5$ range (orange shaded area), corresponding to θ_{HWP} scan angles from 15° and 30° (blue vertical lines), the true zero of $\frac{\Delta I}{I_0}$ for the H7 data is shifted by -0.054. Although this is not expected to impact the NIR focal beam area in the experiments or the subsequent measurement of relative fringe shifts—given that the dipole phase approximately exhibits linear dependence on intensity—it does affect the harmonic amplitude intensity signal. This signal scales with the harmonic order q following the exponential relation $(0.97)^q$, based on the measured NIR peak intensity variation as a function of θ_{HWP} at $\frac{\Delta I}{I_0} = \pm 1$ (Fig. S4).

S1.4. Crystal orientation and polarization characterization measurements of MgO

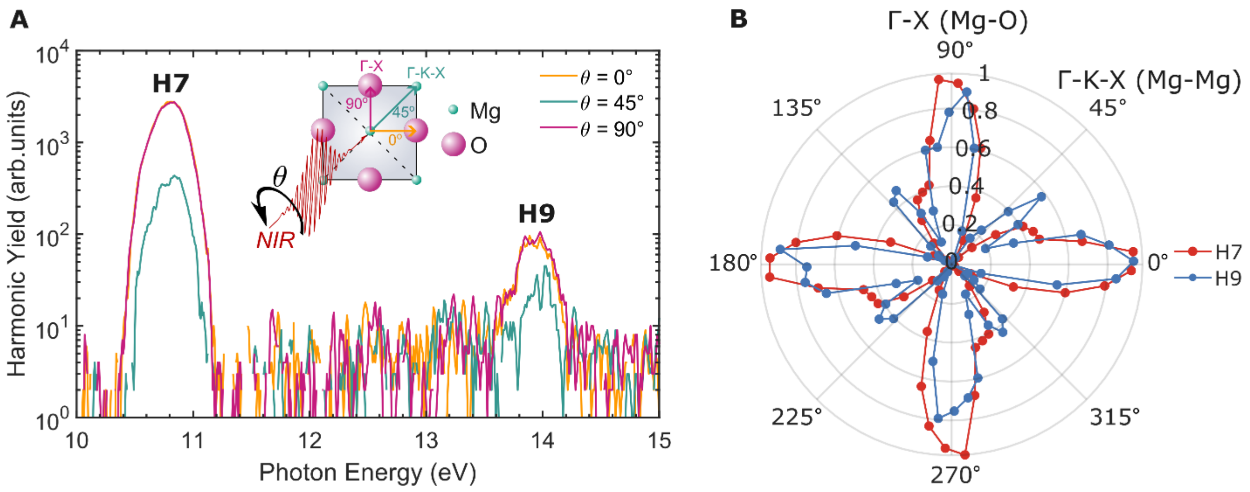

Fig. S6. Crystal orientation-dependent XUV emission measurements in MgO.
(**A**) XUV spectra of harmonic 7 (H7) and harmonic 9 (H9) of the driving NIR field obtained in a (100)-cut MgO crystal, shown for several laser polarization rotation angles θ (0°, 45° and 90°). The harmonic yields are presented on a logarithmic y-scale. The inset illustrates the cubic crystal structure of MgO, highlighting the high-symmetry Γ–X (Mg–O bond) and Γ–K–X (Mg–Mg bond) directions in the crystal lattice and their orientation in the measurements with respect to the NIR driver. (**B**) Normalized XUV intensity signals of H7 and H9 and their angular dependence on θ, with maxima occurring at θ = 0°, 90°, 180°, and 270°, reflecting a 4-fold symmetry associated with the cubic bonding directions in MgO.

In our XUV interferometric experiments, the two replicas of the linearly polarized NIR driving laser pulse were aligned such that their polarization directions were fixed along the Γ–X direction (or Mg–O bond) of the (100)-cut MgO crystal. This alignment was confirmed through crystal orientation-dependent XUV emission measurements. For these experiments, an additional half-wave plate (HWP) was placed before the vacuum HHG chamber to rotate the polarization of a single linearly polarized NIR driver (at peak intensity of 12 TWcm^{-2}), which was focused into the MgO solid in a geometry, as illustrated in the inset of Fig. S6(A). Afterward, the harmonic 7 (H7) and harmonic 9 (H9) signals generated from the MgO by the NIR field were recorded using the same method described in Fig. S1. The HWP was rotated by an angle θ_{HWP} from 0° to 180°,

which corresponds to the laser polarization rotation angle of $\theta = 2\theta_{HWP}$ (i.e., from 0° up to 360°). Fig. S6(**A**) shows the exemplary XUV spectra of H7 and H9 measured at three θ angles (0°, 45°, and 90°), demonstrating the variation in XUV signal strength when probing the crystal orientation from the Γ–X (0°) to the Γ-K-X (45°) and returning to the Γ–X direction. The yields for both harmonics are greater at θ = 0° and 90° (corresponding to the Γ–X or Mg–O bond direction) than at θ = 45° (Γ–K–X), with H7 being about 7 times higher and H9 approximately 3 times higher. The angular dependence of the H7 and H9 intensity signals in panel (**B**), reflecting a 4-fold symmetry associated with MgO's cubic bonding directions, validates the sharper features along the Mg–O bonds, consistent with the observations reported by the Ref. [62]. Consequently, this orientation was selected for the XUV interferometric experiments because it produces the strongest emission signals, which is critical for maximizing signal-to-noise ratio in solid-state HHG.

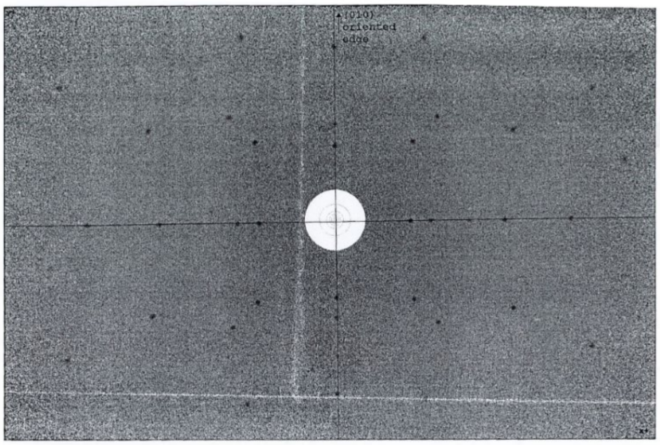

Fig. S7. Laue diffraction analysis of MgO sample.

The image obtained from Laue diffraction crystal orientation analysis of a 100-μm thick MgO crystalline solid used in the experiments. The crystal is cut along the (100) plane with a tolerance of ± 0.3°, and the edge is aligned with (010) within ± 2.0°. The image and the corresponding analysis were supplied by SurfaceNet GmbH.

Note S2. Experimental results

S2.1. Time-dependent harmonic relative phase shift measurements

Fig. S8 shows the temporal far-field XUV interferograms obtained from MgO for harmonic 7 (H7, 10.8 eV) and harmonic 9 (H9, 13.9 eV), measured at equivalent 6 TWcm^{-2} peak intensities at each NIR focus (**A**) with the corresponding intensity profiles of the harmonic fringe patterns (**B**). The recorded interferograms provided insight into the time-dependent relative phase shifts of H7 (refer to the main text) and H9, allowing us to analyze the sensitivity of the birefringent common-path interferometer to the harmonic phase changes. Fig. S9 shows the 2D colour map representing the time-dependent relative phase shift of H9 in MgO, recorded as a function of the time delay, $\Delta\tau$, between the NIR pulse replicas. In the solid-state HHG experiments, the NIR beams peak intensities of several TWcm^{-2} were applied, constrained by the material damage threshold, which was $>$ 12 TWcm^{-2} in this study. Consequently, the emission signals for higher harmonic orders, such as H9 in this instance, exhibit a lower signal-to-noise ratio compared to H7,

as displayed in Fig. S9. Nonetheless, we successfully extracted the harmonic phase shift value for H9 within a 2.7 fs range (800 nm optical cycle), which was found to be $\Delta\varphi_9^\tau = 9*2\pi$ rad. This value corresponds exactly to the harmonic order $q = 9$, confirming the precise synchronization of high-harmonics with the NIR driver in this work.

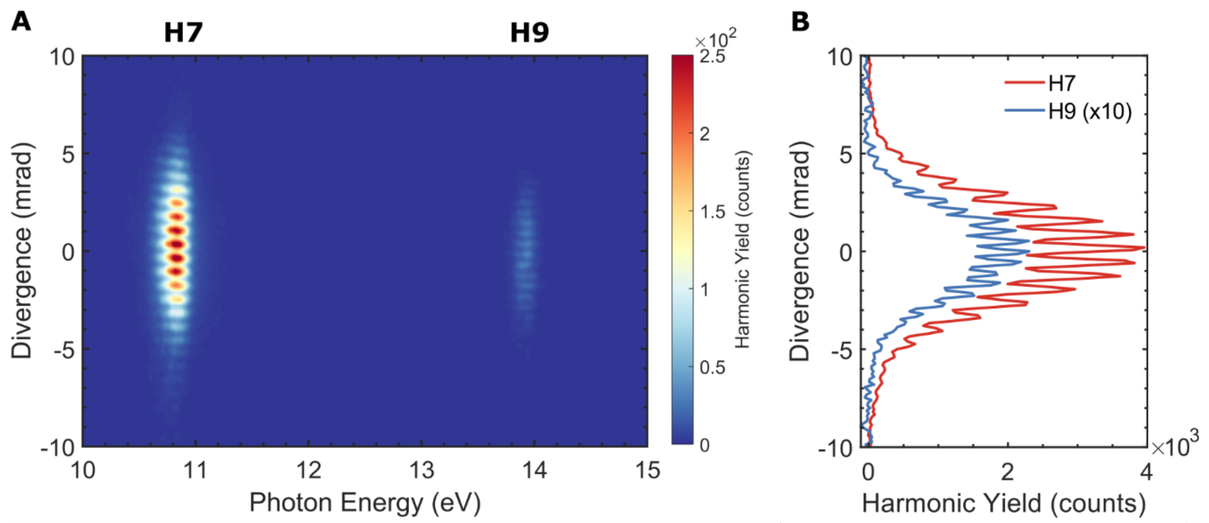

Fig. S8. Temporal XUV interferograms from MgO.

(**A**) Far-field XUV interferograms obtained from MgO for harmonic 7 (H7) and harmonic 9 (H9) at equivalent peak intensities at each NIR focus of 6 TWcm^{-2}. (**B**) Intensity profiles of H7 (red) and H9 (blue) photon-energy-integrated interference fringe patterns. The H9 data is scaled by x10.

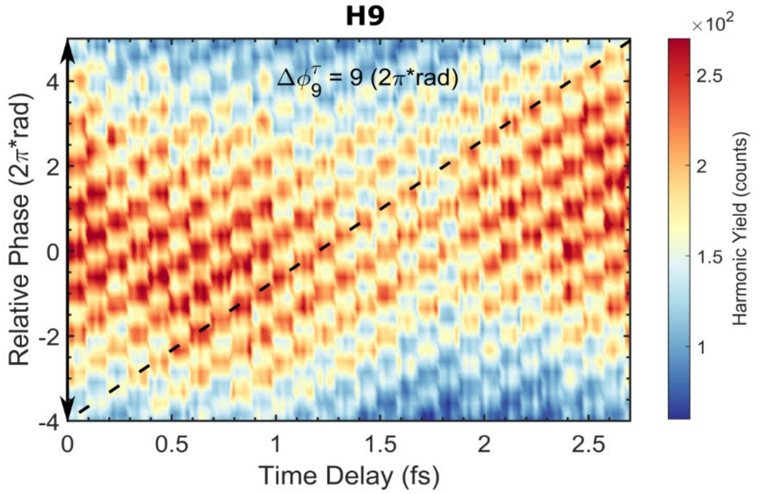

Fig. S9. Time-dependent relative phase shift in MgO for harmonic 9.

Time-dependent relative phase shift, $\Delta\varphi_q^\tau$, in MgO for harmonic 9 (H9), as obtained from the temporal XUV interferograms. The relative fringe shift of H9 within one optical cycle (2.7 fs) of the 800 nm driving laser field is shown with a dashed line. The extracted $\Delta\varphi_9^\tau$ value within this range is shown by the black arrow and is also depicted in the figure.

S2.2. Intensity-induced harmonic relative dipole phase measurements

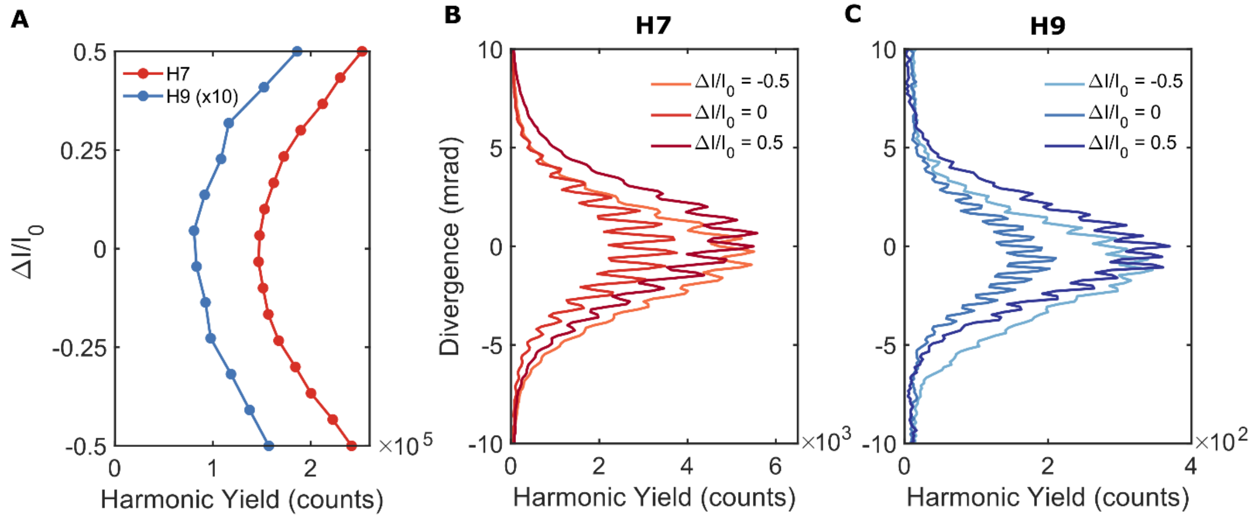

Fig. S10. Intensity-dependent profiles of interference fringes for H7 and H9 in MgO. (**A**) The harmonic yield in MgO for H7 (red) and H9 (blue) as a function of varying relative NIR foci peak intensities, as recorded for $\frac{\Delta I}{I_0} = \pm 0.5$ range in the relative dipole phase measurements. The corresponding profiles of photon-energy-integrated interference fringe patterns for H7 (**B**) and H9 (**C**) at $\frac{\Delta I}{I_0}$ = -0.5, $\frac{\Delta I}{I_0}$ = 0, and $\frac{\Delta I}{I_0}$ = +0.5 (I_0 = 12 TWcm^{-2}).

Fig. S10(**A**) shows the harmonic yield in MgO for H7 and H9 as a function of varying relative NIR foci peak intensities within experimental $\frac{\Delta I}{I_0} = \pm 0.5$ range. Panels (**B-C**) illustrate the intensity-dependent profiles for the photon-energy integrated H7 and H9 interference fringes within MgO at $\frac{\Delta I}{I_0}$ = -0.5 (I_1: 3 TWcm^{-2}, I_2: 9 TWcm^{-2}), $\frac{\Delta I}{I_0}$ = 0 ($I_1 = I_2$ = 6 TWcm^{-2}), and $\frac{\Delta I}{I_0}$ = 0.5 (I_1: 9 TWcm^{-2}, I_1: 3 TWcm^{-2}), highlighting how the driving intensity impact the interference pattern. These data were used in evaluating intensity-induced relative dipole phase shifts, $\Delta\varphi_{q,exp}$, presented in Fig.2(**A-B**) of the main text. First, each fringe line-out of the intensity-dependent interference pattern profile for H7 and H9 was analyzed using a nonlinear least-squares algorithm to fit a Gaussian envelope. The identified local maxima of the individual line-outs (red and blue dots in panel (**A**)), corresponding to the peak spectral intensity values for each harmonic interferogram, were utilized to transform the initial divergence y-axis measured in milliradians (mrad) into the relative fringe shift axis in radians (rad). In this context, the locations of maxima and minima within the fringe pattern signify a 2π rad phase shift, reflecting the distance between two line-out peaks and their relative displacement.

Fig. S11 exemplary displays the raw data measured at $\frac{\Delta I}{I_0}$ = 0 (with equal NIR foci intensities at 6 TWcm^{-2}) fitted to Gaussian envelopes for H7 (left) and H9 (right) in MgO, with the y-axis converted to the relative fringe shift in 2π rad. From the fitted data, the extracted fringe maxima line-out points for H7 and H9 (indicated by the red and blue dots, respectively, in Fig. 2(**A-B**) of

the main text) were then used to perform a linear fit of the intensity-induced relative fringe shifts within the $\frac{\Delta I}{I_0} = \pm 0.5$ range.

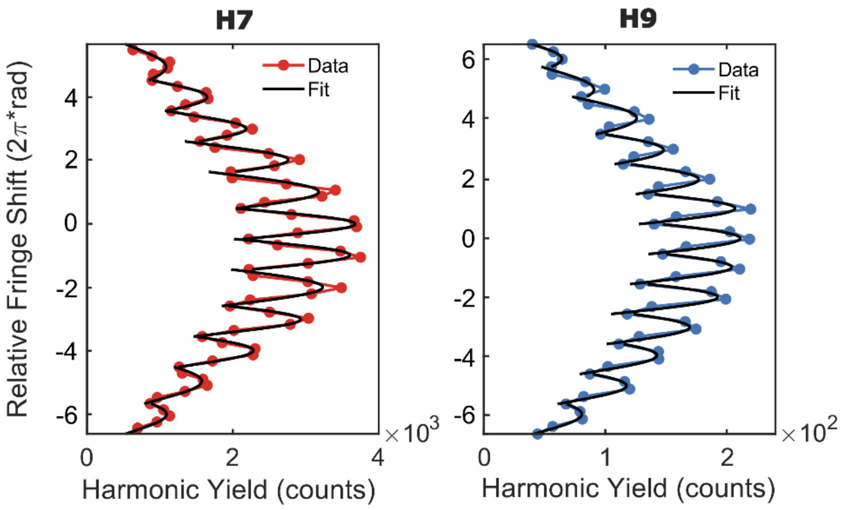

Fig. S11 XUV data fitting procedure.
The raw data line-out fringes from the intensity-dependent XUV interferograms in MgO (dots) presented for the equal NIR foci peak intensities of 6 $TWcm^{-2}$ ($\frac{\Delta I}{I_0} = 0$), fitted to Gaussian envelopes (black lines) for H7 (left panel) and H9 (right panel). The distance between each fringe maxima line-out corresponds to a 2π rad phase shift.

S2.3. Intensity-induced nonlinear phase shift (B-integral) measurements

To isolate and exclude any additional nonlinear effects in MgO that might occur due to the interaction of intense 800 nm laser pulses with the material, we conducted separate intensity-induced phase shift measurements that only recorded the response of the MgO nonlinear medium to the fundamental beam. In the 800 nm interferometric experiments, we ensured that the conditions were identical to those employed in the XUV experiments, up to focusing within the MgO solid. After the NIR beams propagated through the MgO sample, the images of horizontal interference fringes at 800 nm were directly recorded on the CMOS camera, set up in a transmission geometry in the far-field, following the removal of the XUV grating from the beam path (see Fig. S1). The far-field interferograms were recorded as a function of the HWP rotational angle position, corresponding to variations in the NIR peak intensities over the same range used in the XUV experiments ($\frac{\Delta I}{I_0} = \pm 0.5$). Fig. S12 exemplary shows the far-field interferograms of the fundamental driving field obtained from MgO, presented when the peak intensities of the NIR beams were matched for each focus $I_1 = I_2 = 6$ $TWcm^{-2}$ ($\frac{\Delta I}{I_0} = 0$) (**A**), and when all the intensity was placed into a single beam, where $I_1 = 12$ $TWcm^{-2}$ and $I_2 = 0$ $TWcm^{-2}$ ($\frac{\Delta I}{I_0} = 1$) (**B**).

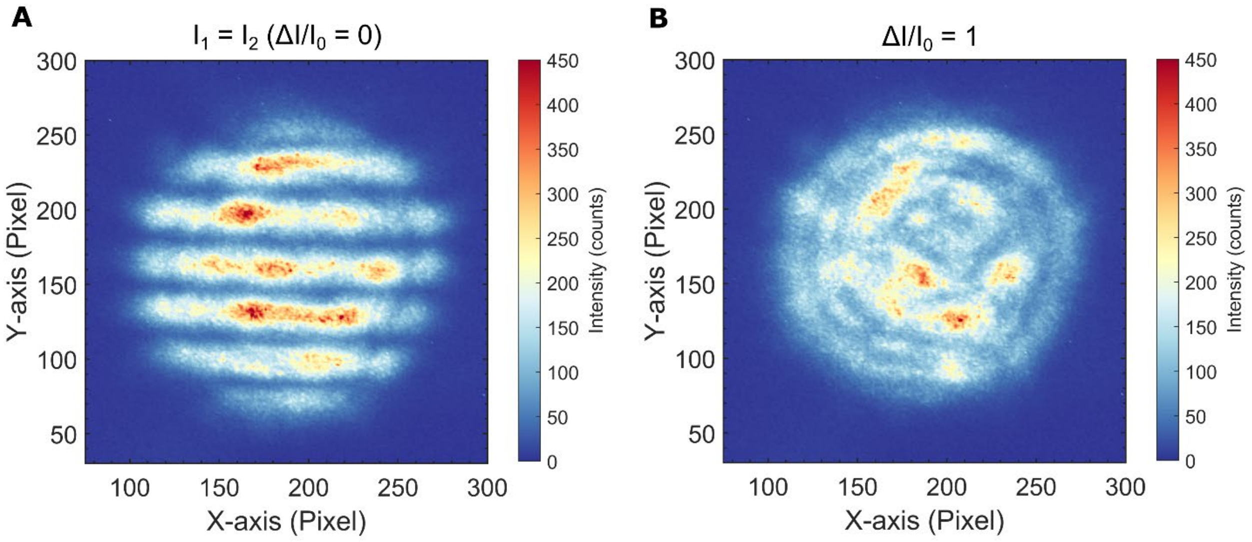

Fig. S12 Intensity-induced interferograms for 800 nm emission from MgO.
The far-field interferograms for the fundamental 800 nm driving field obtained from MgO during the intensity-dependent (B-integral) measurements, presented for two scenarios: (**A**) where the NIR beams have equal peak intensities of 6 TW/cm² at each focus ($\frac{\Delta I}{I_0}$ = 0), and (**B**) where all the intensity is combined into one beam, I_1 = 12 TWcm^{-2} and I_2 = 0 TWcm^{-2} ($\frac{\Delta I}{I_0}$ = 1).

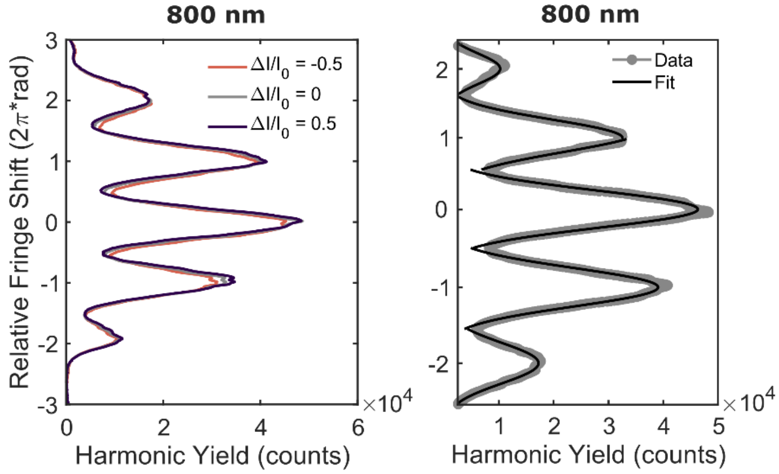

Fig. S13 800 nm (B-integral) data fitting procedure.
The intensity-dependent profiles of interference fringe patterns at 800 nm obtained from MgO, presented for the NIR foci peak intensities of $\frac{\Delta I}{I_0}$ = -0.5, $\frac{\Delta I}{I_0}$ = 0, and $\frac{\Delta I}{I_0}$ = +0.5 (left panel). The selected line-out data of fringes (dots) from the nonlinear phase shift measurements (refer to Fig. 2C in the main text), displayed for 6 TWcm^{-2}, along with Gaussian envelopes fitted to the individual fringe line-outs (black line), shown in the right panel.

The left panel in Fig. S13 displays the intensity-dependent profiles of interference fringe patterns at 800 nm in MgO, presented for the NIR foci peak intensities of $\frac{\Delta I}{I_0}$ = -0.5 (I_1: 3 TWcm^{-2},

I_2: 9 TWcm^{-2}), $\frac{\Delta I}{I_0}$ = 0 (I_1 = I_2 = 6 TWcm^{-2}), and $\frac{\Delta I}{I_0}$= +0.5 (I_1: 9 TWcm^{-2}, I_1: 3 TWcm^{-2}). While the relative fringe shift for varying fundamental intensities is not as prominent as observed in the XUV data (see Fig. S10(**B-C**)), it gains importance when we recognize that this phase shift scales linearly with the harmonic order. Analogously to the XUV data analysis, each fringe line-out of the intensity-dependent interference pattern profile at 800 nm was fitted to the Gaussian envelope, as exemplary shown in the right panel of Fig. S13. Using the fitted data, the extracted fringe maxima line-out points (shown as gray dots in Fig. 2(**C**) of the main text) were employed to carry out a linear fit of the intensity-induced nonlinear phase shifts, $\Delta\varphi_{800}$, across the identical measured intensity range of $\frac{\Delta I}{I_0} = \pm 0.5$ as in the harmonic data. This enabled us to quantify the $\Delta\varphi_{800}$ in our XUV interferometric experiments and to subtract the B-integral contribution at 800 nm in MgO.

Note that the spatial averaging effects are significant for the phase contribution of the fundamental, and negligible for the harmonics. The inclusion of spatial averaging for the fundamental is described in detail by Ref. [52]. The reason for this difference is that the phase scales approximately linearly with intensity, and the amplitude roughly follows the power law of the harmonic order (or linearly for the fundamental). In other words, the harmonic phase contribution at lower intensities in the focus area is negligible as the corresponding harmonic amplitude is very small. We simulated the effect to be well below a 10% correction.

We also estimated propagation effects in MgO, such as absorption, and dispersion of the generated harmonics that may influence our calibration approach. The key quantities to assess the contribution of propagation effects are the coherence length,

$$L_c = \frac{\pi}{\Delta k} = \frac{\pi}{(k_q - qk_1)} = \frac{\lambda_0}{2q(n_q - n_1)}, \tag{S4}$$

where $k_q = \frac{2\pi n_q}{\lambda_q}$ is the wave vector of a harmonic order q, λ_0 and λ_q are the fundamental and harmonic wavelengths; and the absorption length,

$$L_a = \frac{\lambda_q}{4\pi k}, \tag{S5}$$

with n and k are the real and imaginary parts of the refractive index, respectively. The coherence length defines where phase mismatch causes destructive interference, while the absorption length indicates where the light is reabsorbed. Typically, efficient HHG requires L_c to be larger than L_a; if L_c is much greater, phase matching effects are negligible, especially in thin HHG targets, for instance, in gas jets in gas-phase HHG.

We computed these quantities using the complex refractive index data from Ref. [53], validated with atomic scattering factors from the CXRO database [54]. We found that for H7 (114.8 nm), $n = 1.83$, $k = 1.22$, yielding $L_c \approx 530$ nm and $L_a \approx 7.5$ nm. For H9 (89.2 nm), $n = 1.04$, $k = 0.97$,resulting in $L_c \approx 65$ nm and $L_a \approx 7.3$ nm. These results demonstrate that the absorption length is extremely short (~7 nm), significantly smaller than the coherence length (between 65 nm and 115 nm), indicating that absorption dominates the propagation effects. Consequently, the generated harmonics are absorbed before any substantial phase-matched buildup can occur. This phenomenon is common in XUV emission from solids in HHG, where very short absorption lengths hinder high efficiency. However, this characteristic can be advantageous for using XUV HHG as a tool to probe microscopic effects in solids, as we do in this study.

S2.4. Additional studies of the intensity-induced harmonic dipole phases in MgO:Cr vs. MgO

We performed additional XUV interferometric experiments using a chromium-doped MgO (MgO:Cr) as the solid HHG target in the dipole phase measurements. The MgO:Cr solid was double-side polished, 100 µm-thick, with its (100)-cut plane oriented as in the pure MgO sample employed in this work (both purchased from SurfaceNet GmbH). The doping concentration of the Cr atoms in the MgO:Cr was 1000 ppm (or 0.1% defect concentration), introduced into the MgO crystal during triarc plasma growth by the supplier. The intensity-induced dipole phase experiments with MgO:Cr were conducted under conditions similar to those with pure MgO described in this work, except for the vertical separation distance between the NIR foci, which was set to 420 µm given a focal spot size of 50 µm (FWHM) for each beam. We deliberately increased this separation to assess whether a separation of ≥4 times the beam spot diameter influences the dipole phase measurements and outcomes. During the experiments, the MgO:Cr sample was mounted beneath the undoped MgO sample on the same three-dimensional manipulator, enabling successive XUV interferogram measurements from both samples by vertically shifting the entire sample-holder assembly within the HHG interaction region.

Fig. S14 exemplary shows the far-field XUV interferograms obtained from pure MgO (**A**) and MgO:Cr (**C**), with driving NIR beams having equal peak intensities of 6 $\mathrm{TWcm^{-2}}$ at their foci. The fringe pattern intensity profiles for harmonic 7 (H7) are displayed in panels (**B**) for MgO and (**D**) for MgO:Cr. From the figure, it is evident that the divergence increased and the fringe spacing in the recorded XUV interference patterns decreased compared to the data in Fig. S10. Moreover, the interference pattern of harmonic 9 (H9) for both samples is barely discernible, preventing further analysis of its interference fringes. This occurs because the fringe separation in the far-field on the detector plane is directly proportional to the driving wavelength and inversely proportional to the distance between the two focal points, so higher harmonic orders with shorter wavelengths produce smaller fringe spacings. Consequently, achieving reliable XUV interferometric dipole phase measurements require careful consideration of the two NIR beams' separation in the focal plane. The separation should be as small as possible to facilitate detection of the far-field XUV interference patterns, yet sufficiently large to prevent fundamental frequency oscillations that arise when the foci are too close. This can be checked and refined by performing interferometric measurements of the temporal relative phase shifts of XUV harmonics, as proposed in this work.

Fig. S15 shows the 2D colour maps of the intensity-induced relative fringe shifts associated with a change in the dipole phase, $\Delta\varphi_{7,exp}$, for H7 in the pure MgO (**A**) and MgO:Cr (**B**) recorded as a function of the varying relative peak intensities of the NIR pulse replicas for the foci separation of 420 µm. The $\Delta\varphi_{7,exp}$ values were obtained through linear fits to the fringe shifts over the $\frac{\Delta I}{I_0} = \pm 0.5$ peak intensity range. The experimental $\Delta\varphi_{7,exp}$ values are determined to be 0.54 ± 0.11 (2π rad) for MgO and 0.49 ± 0.09 (2π rad) for MgO:Cr. The total B-integral contribution to the H7 phase in the 100 µm-thick MgO:Cr sample was found to be the same as in the undoped MgO (see Fig. 2C of the main text), with a value of $\Delta\varphi_{7,B}$ = 0.26 ± 0.07 (2π rad). After subtracting the B-integral from the experimentally measured fringe shifts, the resulting relative dipole phases for H7 are $\Delta\varphi_7$ = 3.52 ± 1.17 rad in MgO and $\Delta\varphi_7$ = 3.33 ± 1.01 rad in MgO:Cr. Fig. S16 displays a comparison of the relative dipole phase results in MgO (**A**) and MgO:Cr (**B**) for H7 within the $\frac{\Delta I}{I_0} = \pm 0.5$ intensity range. The results demonstrate that the dipole phase of H7 exhibits a linear dependence on intensity that is about 0.19 rad less in Cr-doped MgO than in undoped MgO. Still, this difference is considered minor given the experimental uncertainties.

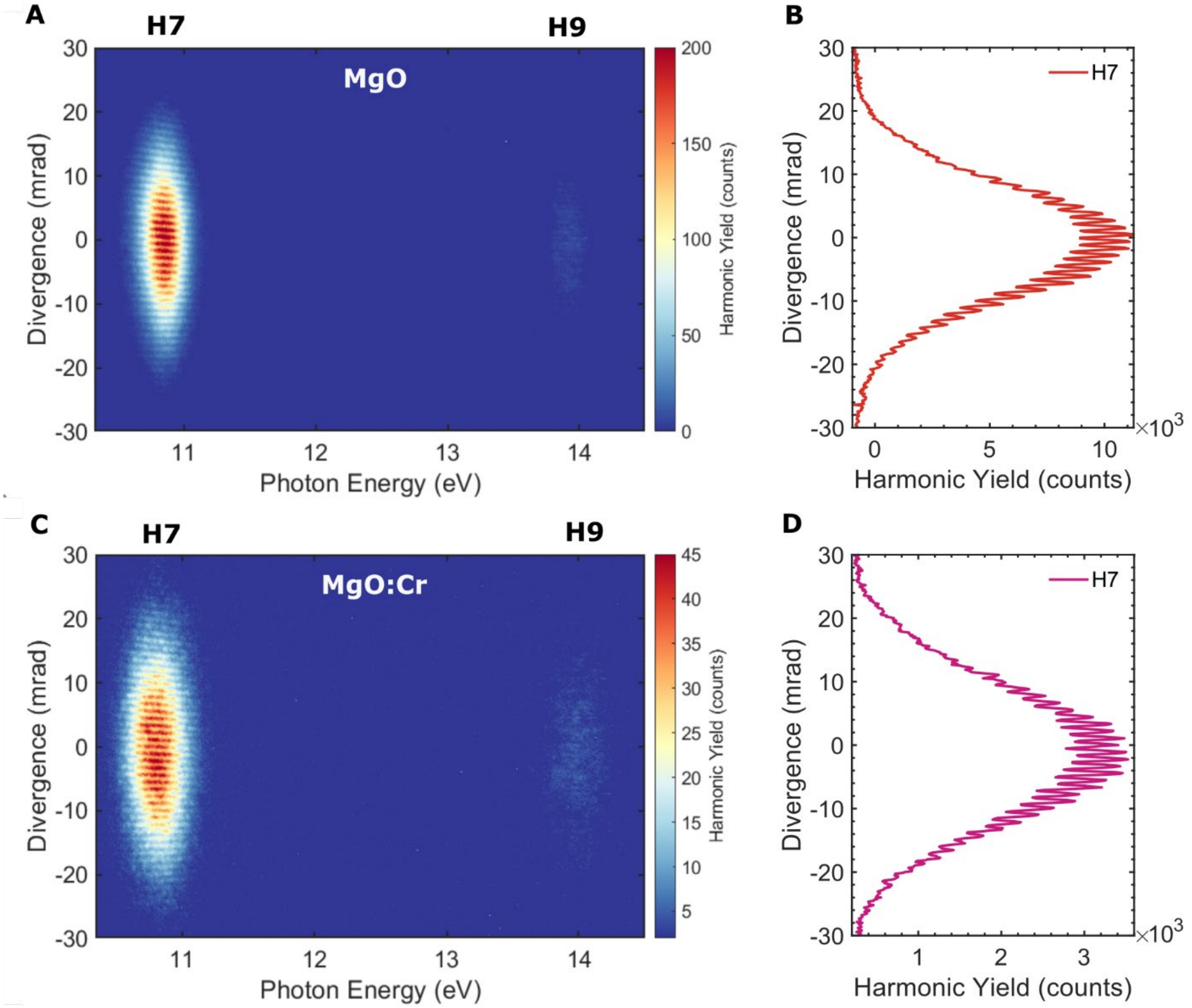

Fig. S14 Intensity-induced XUV interferograms from MgO and MgO:Cr.

Far-field XUV interferograms obtained from MgO (**A**) and MgO:Cr (**C**), recorded for the NIR foci separation distance of 420 μm and peak intensities of 6 $TWcm^{-2}$ at each focus. The harmonic orders (H7 and H9) refer to the odd multiples of the 800 nm driver. The intensity profiles of photon-energy-integrated H7 interference fringe patterns for MgO (**B**) and MgO:Cr (**D**), correspondingly.

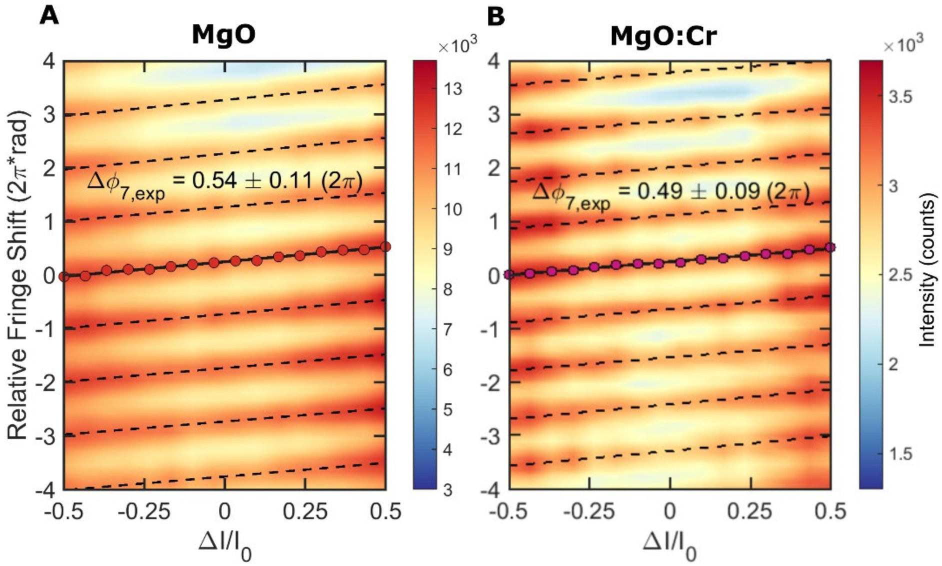

Fig. S15 Intensity-induced relative fringe shifts for H7 emission from MgO and MgO:Cr. The relative fringe shifts, $\boldsymbol{\Delta\varphi_{7,exp}}$, for harmonic 7 (H7) in MgO (**A**) and MgO:Cr (**B**) measured along the Γ–X (Mg–O bond) direction, as a function of the normalized intensity difference of the NIR pulse pair $\boldsymbol{\Delta I/I_0}$ ($\boldsymbol{I_0}$ = 12 TWcm^{-2}) for the foci separation of 420 μm. $\boldsymbol{\frac{\Delta I}{I_0} = \pm 0.5}$ range corresponds to variations in NIR foci peak intensities from 3 to 9 TWcm^{-2} in one arm and inversely in the other; equal intensities (6 TWcm^{-2}) occur at $\boldsymbol{\frac{\Delta I}{I_0} = 0}$. The $\boldsymbol{\Delta\varphi_{7,exp}}$ values, were determined for the local fringe maxima for MgO (red dots) and MgO:Cr (pink dots) through linear fits of the fringe shifts (dashed lines) over the $\boldsymbol{\frac{\Delta I}{I_0} = \pm 0.5}$ range. The $\boldsymbol{\Delta\varphi_{7,exp}}$ (in radians), corrected for the B-integral effect at 800 nm for H7 are depicted in Fig. S16.

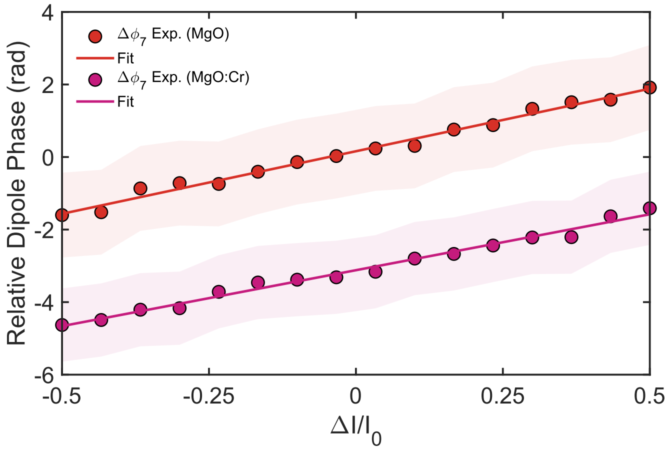

Fig. S16 Intensity-induced relative dipole phase results for H7 ($\boldsymbol{\Delta\varphi_7}$) in MgO and MgO:Cr. Comparison of experimental relative dipole phase results for H7 ($\Delta\varphi_7$) in MgO (red dots) and MgO:Cr (pink dots) along the Γ–X (Mg–O bond) direction, determined within the measured intensity range of $\frac{\Delta I}{I_0} = \pm 0.5$ and incorporating the harmonic-weighted B-integral contributions. The reference point at $\frac{\Delta I}{I_0} = 0$ (I_0 = 12 TWcm^{-2}) indicates equal intensities of 6 TWcm^{-2} in the NIR

foci. The solid red and pink lines represent the linear fits of the intensity-induced relative fringe shifts in MgO and MgO:Cr, respectively. The corresponding shaded error bars represent the $\pm 2\sigma$ uncertainties derived from linear fits to the experimental fringe patterns.

Note S3. Simulation results

S3.1. Multi-band (full electronic band structure) numerical calculations

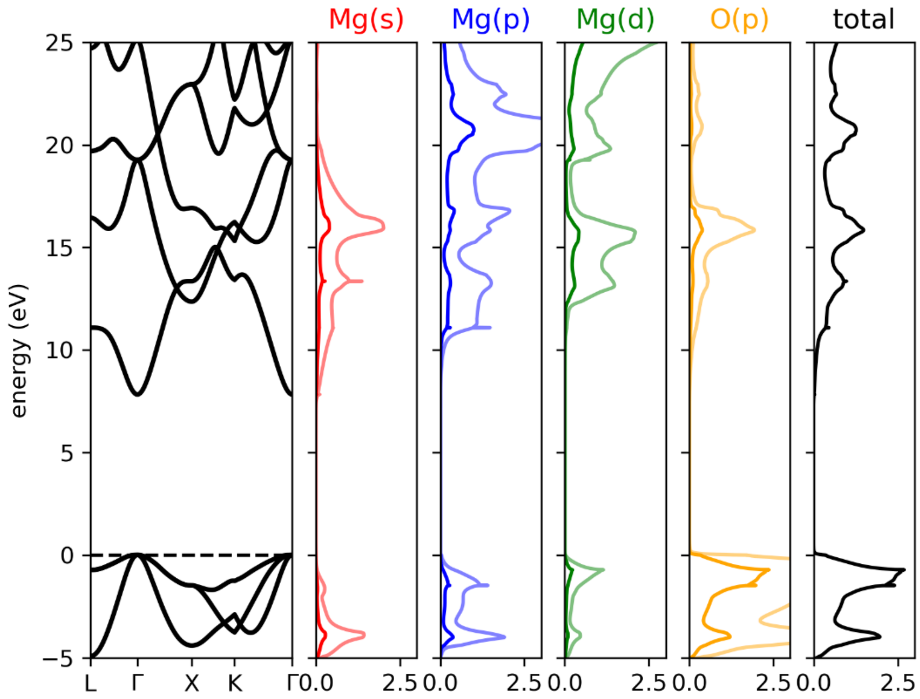

Fig. S17 Orbital- and band-resolved multi-band numerical calculation results for MgO. The electronic band structure (left panel) and orbital-resolved projected density of states (PDOS) (right panels) in MgO. The zero-energy level is set to the VB maximum (dashed line). The direct band gap is 7.8 eV at the Γ-point. Four PDOS plots for the magnesium Mg (s, p, d) and oxygen O (p) orbitals are depicted in different colors, with the total PDOS represented in black. The two lines per panel are original data and a zoom (x5) thereof.

S3.1.1 Additional simulation results for the Γ–X crystal direction (Mg–O bond)

Fig. S18 shows the additional results for the intensity-induced relative dipole phases, $\Delta\varphi_5$, and $\Delta\varphi_{11}$ simulated for H5 (8 eV) and H11 (17 eV) along the Mg–O bond (Γ–X crystal direction) in MgO, using the multi-band model. A comparison of the intensity dependence of $\Delta\varphi_5$ for H5 and $\Delta\varphi_{11}$ for H11, versus H7 ($\Delta\varphi_7$) and H9 ($\Delta\varphi_9$) is shown in panel (**A**). Normalized populations for the computed harmonics (H5-H11) within the same intensity range are presented in panel (**B**). The Mg (s, p, d) and O (p) orbital contributions to the relative dipole phase of H5 and H11 are visualized in panels (**C-D**), using the calculated Fourier phases and amplitudes.

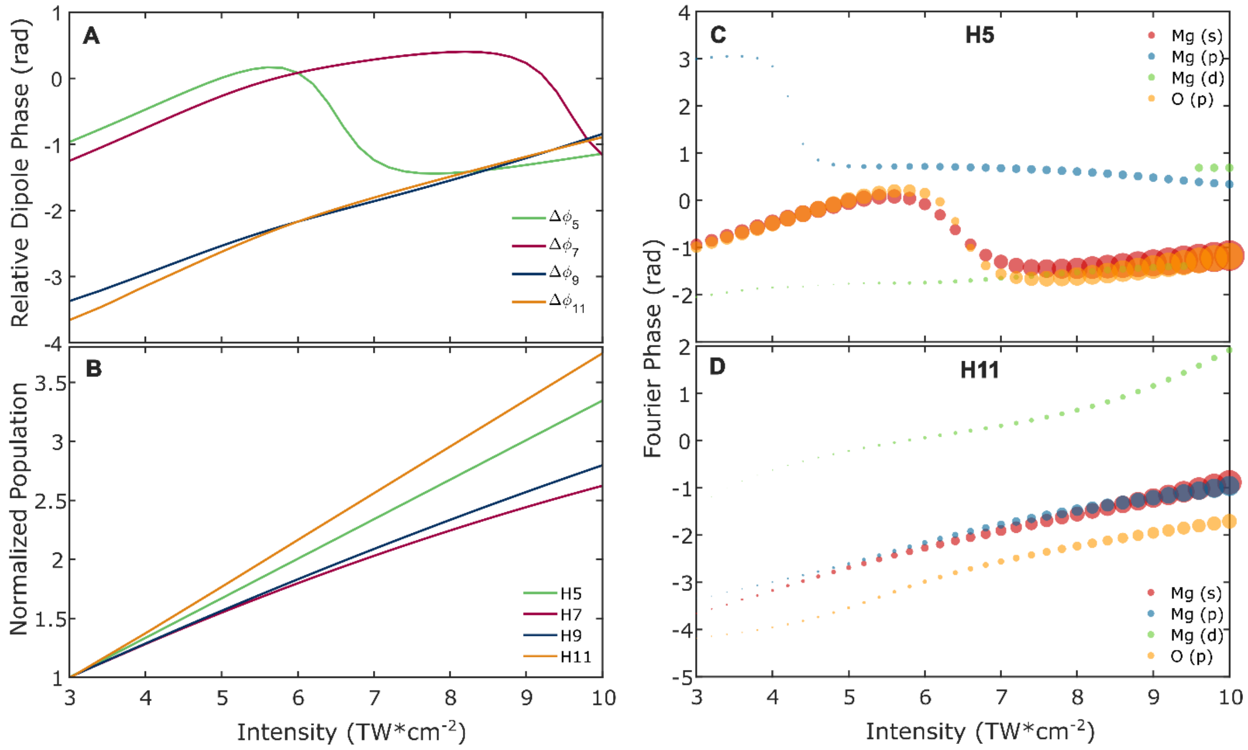

Fig. S18 Multi-band numerical simulations for the Mg–O bond (Γ–X direction).
(**A**) Simulated relative dipole phase results for harmonic 5 ($\Delta\varphi_5$) and harmonic 11 ($\Delta\varphi_{11}$) in MgO, using the multi-band model, in comparison with H7 ($\Delta\varphi_7$) and H9 ($\Delta\varphi_9$) results presented in the main text, along the Mg–O bond (Γ–X direction), within the 3–10 TWcm^{-2} intensity range. For optimal visualization, the $\Delta\varphi_5$ and $\Delta\varphi_{11}$ curves are vertically offset, with the reference point set at 6 TWcm^{-2}. (**B**) Normalized population of the corresponding harmonics (H5-H11) over the same intensity range, as obtained from the calculations. (**C-D**) The Fourier phases and amplitudes of the Mg (s, p, d) and O (p) orbitals for harmonic 5 (H5) and harmonic 11 (H11), respectively, as obtained from the orbital-based multi-band analysis. The size of each circle indicates the magnitude of its Fourier amplitude, with distinct colors representing different orbitals.

S3.1.2 Simulation results for the Γ–K–X crystal direction (Mg–Mg bond)

Fig. S19 shows the simulated intensity-induced relative dipole phases (**A**) and the corresponding populations (**B**) for harmonics 5 ($\Delta\varphi_5$), 7 ($\Delta\varphi_7$), 9 ($\Delta\varphi_9$), and 11 ($\Delta\varphi_{11}$) in MgO along the Mg–Mg bond (Γ–K–X crystal direction), using a multi-band methodology analogous to that employed in the simulations presented for the Mg–O bond. The results demonstrate that compared to the Mg-O bond (Γ–X direction), the intensity-dependence of the $\Delta\varphi_{11}$ along the Mg-Mg bond (Γ–K–X) is strongly deviates for increasing intensity. Our interpretation is as follows: Along the Γ–X direction, both H9 and H11 originate from transitions involving the second CB, resulting in similar dipole phase dependence on intensity (see PDOS in Fig. S17). In contrast, along the Γ–K–X direction, H9 corresponds to a region within the band gap, whereas H11 again involves the second CB, leading to a different dipole phase dependence on intensity. The orbital contributions (Mg (s, p, d) and O (p)) to the relative dipole phase of H5, H7, H9, and H11 in MgO along the Mg–Mg bond are illustrated in Fig.S20(**B-D**). Our results demonstrate that at higher laser

intensities, the dipole phase for H11 is primarily governed by contributions from Mg (s), Mg (p), and Mg (d) orbitals, collectively shaping the overall dipole phase along the Mg–Mg bond.

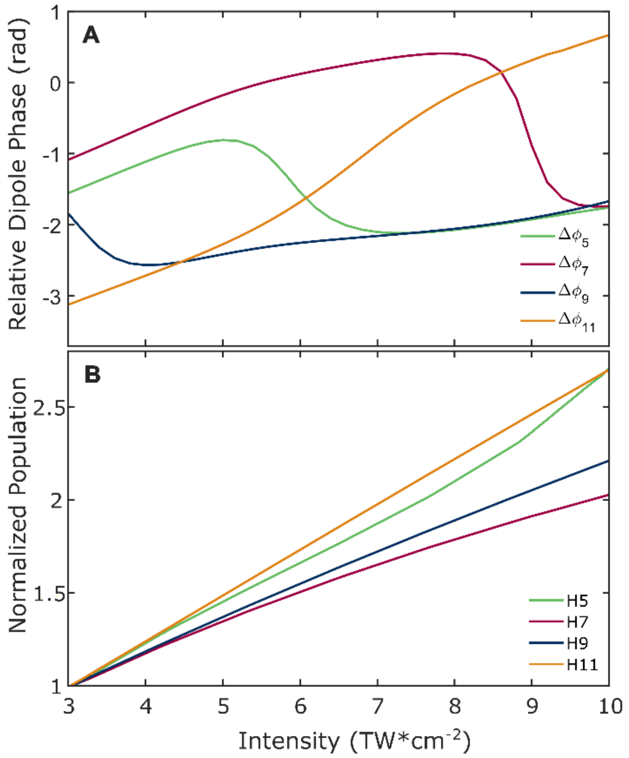

Fig. S19 Multi-band numerical simulations for the Mg–Mg bond (Γ–K–X direction).

(**A**) Simulated relative dipole phase results for harmonics 5 ($\Delta\varphi_5$), 7 ($\Delta\varphi_7$), 9 ($\Delta\varphi_9$), and 11 ($\Delta\varphi_{11}$) in MgO as a function of 800 nm driving laser intensity aligned along the Mg–Mg bond (Γ–K–X direction), using the multi-band model. (**B**) Normalized population of the corresponding harmonics (H5-H11) within the same 3–10 TWcm^{-2} intensity range, as obtained from the calculations.

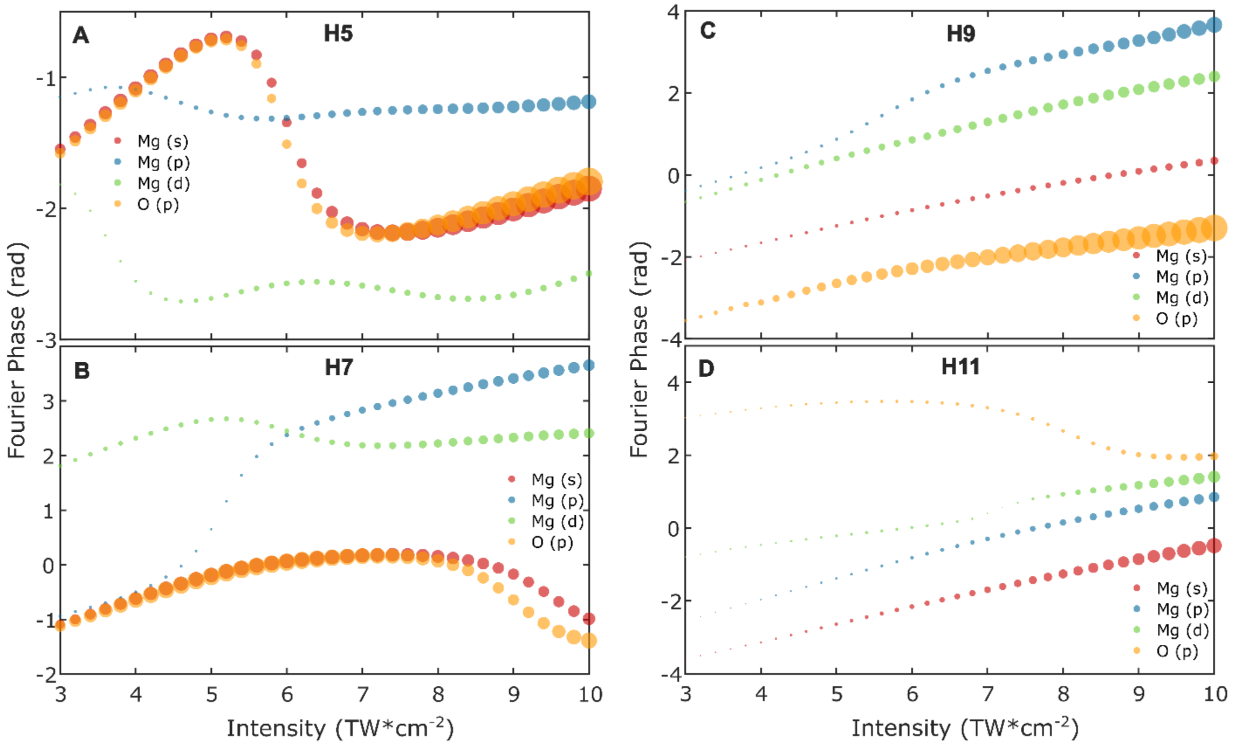

Fig. S20 Multi-band numerical simulations for the Mg–Mg bond (Γ–K–X direction).
The Fourier phases and amplitudes of the Mg (s, p, d) and O (p) orbitals as a function of the 800 nm laser intensity for H5 (**B**), H7 (**B**), H9 (**C**), and H11 (**D**), as derived from the multi-band simulations. The size of each circle indicates the magnitude of its Fourier amplitude, with distinct colors representing different orbitals.

S3.2. Two-band analytical and numerical calculations

For the two-band analytical and numerical calculations of the dipole phase the electronic band structure of MgO with a cubic (100) crystal structure was utilized, as described in Ref. 62. The corresponding conduction (**A**) and valence (**B**) bands of MgO, as well as a cross-section of the bands along the two crystal directions of high symmetry: Γ–X (Mg–O bond) and Γ–K–X (Mg–Mg bond) are shown in Fig. S21. For both high symmetry directions, we assessed the dependence of the dipole phase on the fundamental 800 nm laser intensity. Panel (**A**) in Fig. S22 displays the analytically simulated intensity-dependent relative dipole phase results for harmonics 7 ($\Delta\varphi_7$) and 9 ($\Delta\varphi_9$), presented for both short (s) and long (l) electron trajectories, generated by the 800 nm laser pulses along the Γ–K–X crystal direction (Mg-Mg bond) in MgO. Panel (**B**) displays the corresponding results for the Γ–X direction (Mg-O bond), which are also addressed in the main text. According to the simulated analysis, the intensity-dependent dipole phase behavior of H7 exhibits similarities for both crystal directions involved in harmonic generation from MgO, with emissions initiating at about 5 TWcm^{-2} peak intensity. For H9, harmonic emission begins at 11 TWcm^{-2} for the Γ–K–X crystal direction and at 15 TWcm^{-2} for the Γ–X direction. The relative dipole phase shift for the Γ–K–X direction is more pronounced compared to the other high-symmetry crystal direction in the same intensity range. Moreover, the larger relative dipole phase change is observed for the calculated long electron trajectories when compared to the short ones.

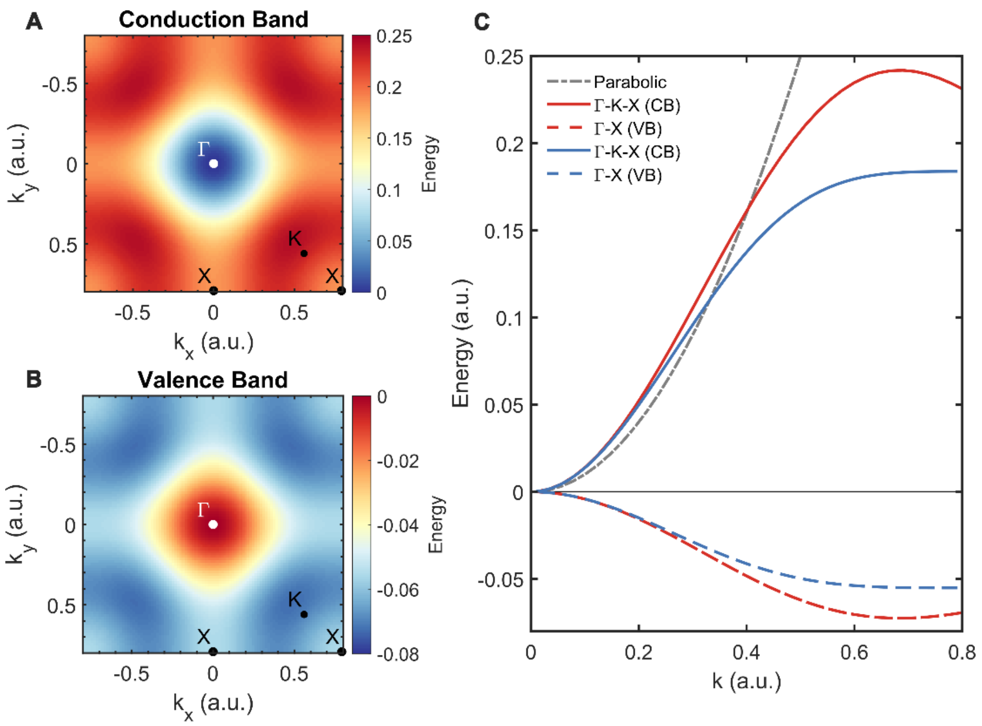

Fig. S21 MgO crystal and band structure within two-band analytical and numerical models.

The crystal structure of MgO (100) in a momentum space for the conduction band (CB) (**A**) and valence band (VB) (**B**). Panel (**C**) illustrates a cross-section of the bands along the Γ–X (Mg–O bond) and Γ–K–X (Mg–Mg bond) crystal directions, along with a reference parabola. For better visualization, the band gap has been set to zero.

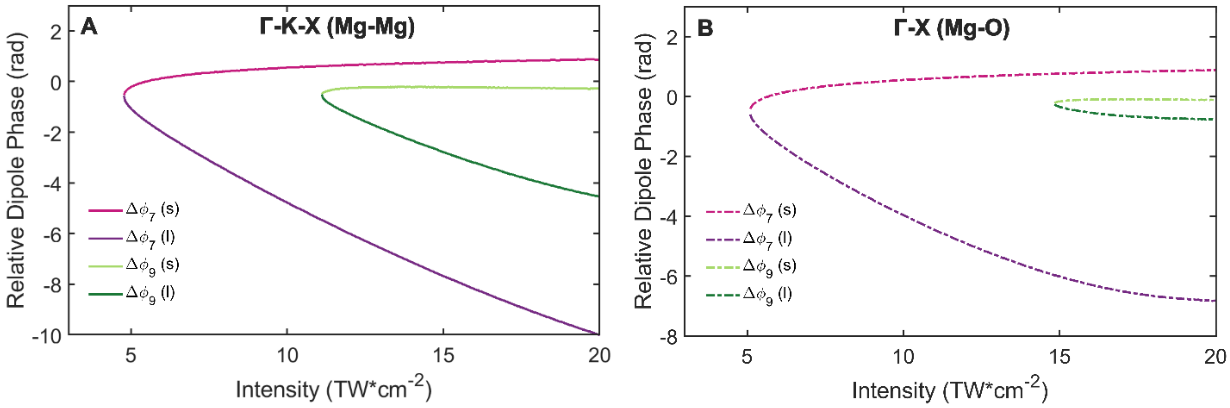

Fig. S22 Two-band analytical simulations for the Γ–X / Γ–K–X directions.

The calculated relative dipole phase as a function of the peak intensity with the 800 nm driver for harmonics 7 ($\Delta\varphi_7$), and 9 ($\Delta\varphi_9$), shown along the Γ–K–X (**A**) and Γ–X (**B**) crystal directions in MgO, derived from the two-band analytical semi-classical model. The figures highlight the intensity-dependent nature of dipole phase behavior for both short (s) and long (l) laser-driven electron trajectories, while also pinpointing the importance of the high-symmetry crystal direction involved in the generation of high-harmonic emission in MgO solid.

S3.3. Dipole phase values comparison in MgO: simulations vs. Ref. [35] reflection data

We compare the simulated intensity-induced relative dipole phase results for H7 ($\Delta\varphi_7$) and H9 ($\Delta\varphi_9$) obtained from the analytical (two-band) and numerical (two-band and multi-band) models developed in this work, with the experimental data reported by Lu *et al.* [35] for MgO along the Mg–O bond (Γ–X crystal direction). These experimental results were obtained in reflection geometry, using similar driving laser parameters (800 nm central wavelength, 50 fs pulse duration, 1 kHz repetition rate) and NIR beam focal spot diameters of 50 μm (FWHM), comparable to the interferometric XUV measurements conducted in transmission geometry described in this study.

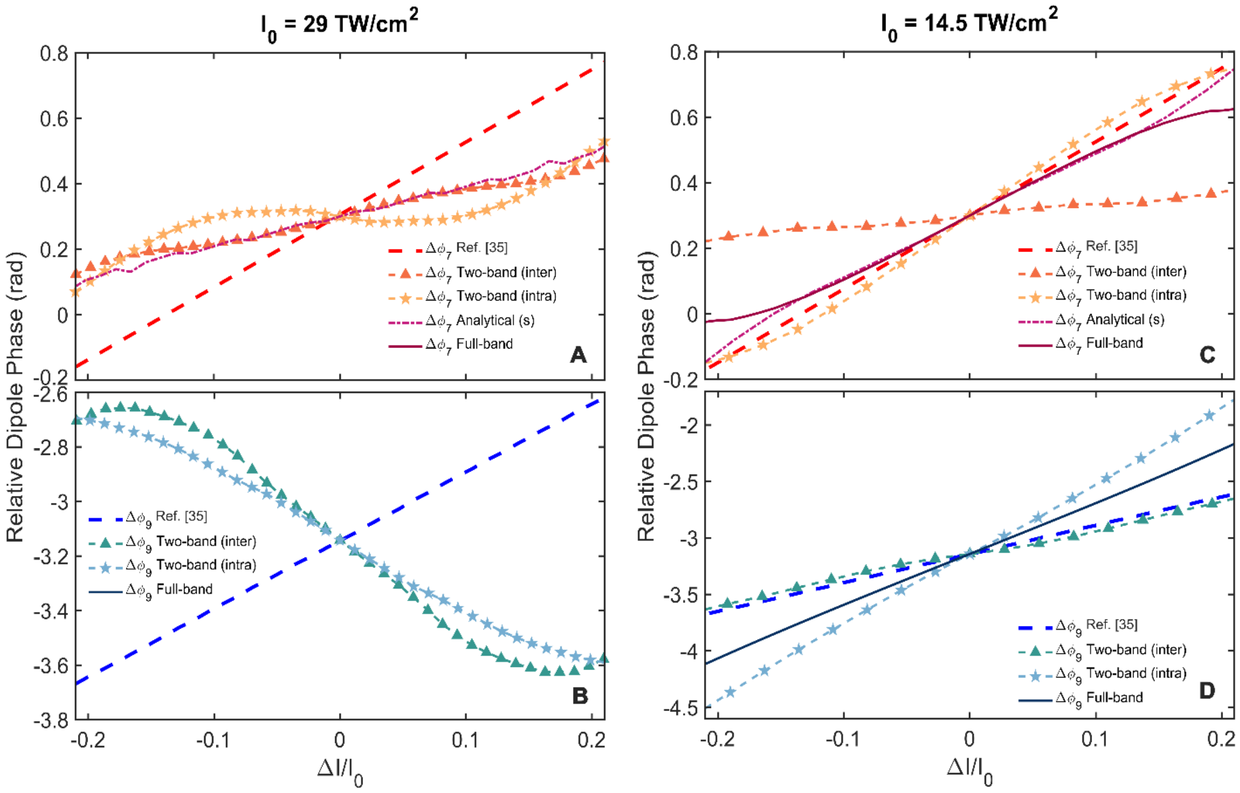

Fig. S23 Comparison of two-band and multi-band theoretical simulations with reflection measurements from Ref. [35] for harmonics 7 and 9 relative dipole phase results in MgO. (**A-B**) Experimental $\Delta\varphi_7$ (red dashed line) and $\Delta\varphi_9$ (blue dashed line) results obtained in reflection geometry with the 800 driver within the intensity range of $\Delta I/I_0 = \pm\,0.21$, as reported in Ref. [35] for MgO along the Γ–X (Mg–O bond) direction, compared to the analytical semi-classical and numerical two-band and multi-band simulations in this work, with the reference point ($\Delta I/I_0 = 0$) set at I_0 = 29 TWcm^{-2} for the computed results. (**C-D**) The same data (panels **A-B**), now shown for the theoretical curves with the $\Delta I/I_0$ reference point set at the lower driving peak intensities of I_0 = 14.5 TWcm^{-2} (half of the previous panels). For clarity, the experimental curves from Ref. [35] are vertically offset so that their reference point aligns at $\Delta I/I_0 = 0$ with the simulations.

Fig. S23(**A-B**) displays the results of the theoretical relative dipole phase simulations in comparison to the experimental values reported in Ref. [35], which are within their measured $\Delta I/I_0 = \pm 0.21$ range at the peak intensity of I_0 = 29 TWcm^{-2} inside the MgO sample (where at $\Delta I/I_0 = 0$, $I_1 = I_2 = 14.5$ TWcm^{-2}). For an exact comparison, the fringe shifts for H7 ($\Delta\varphi_7$ = 0.15*2π rad) and H9 ($\Delta\varphi_9$ = 0.17*2π rad) measured by Lu *et al.* were converted into radians, yielding in $\Delta\varphi_7$ (Ref. [35]) = 0.94 rad and $\Delta\varphi_9$ (Ref. [35]) = 1.07 rad, respectively. As shown in the figure, the experimental values of $\Delta\varphi_7$ and $\Delta\varphi_9$ from Ref. [35] deviate significantly from all the theoretical predictions. Yet, if we assume that Lu *et al.* overestimated the driving peak intensity by a factor of 2 (with I_0 ~14.5 TWcm^{-2} and $I_1 = I_2 = 7.25$ TWcm^{-2}), which closely matches our experimental total peak intensity in MgO of 12 TWcm^{-2}, the $\Delta\varphi_7$ and $\Delta\varphi_9$ reported in Ref. [35] align almost perfectly with the theoretical predictions computed for this varied $\Delta I/I_0$ intensity range, as illustrated in panels (**C-D**) of Fig. S23. This highlights the importance of accurately defining the peak intensity inside the sample, I_0, as it directly affects the dipole phase results at the $\Delta I/I_0$ reference point, particularly in the context of theory-experiment comparisons.

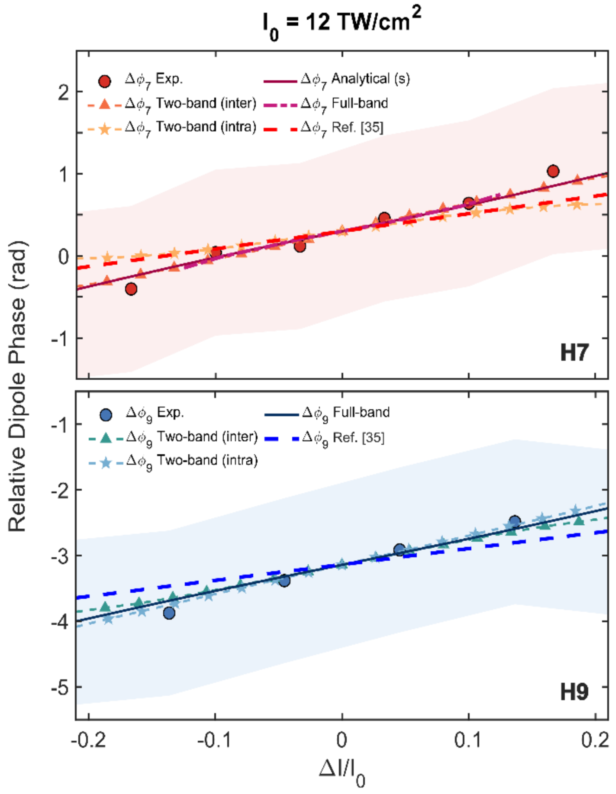

Fig. S24 Experimental and theoretical relative dipole phase results obtained in this work compared with reflection data from Ref. [35] for harmonics 7 and 9 in MgO.

To delve into the differences and similarities between the dipole phase values from our transmission-based experimental arrangement and those in Lu et al.'s reflection geometry study—both using the same wavelength and harmonic orders—we conducted a comparative analysis of $\Delta\varphi$ for H7 and H9 across the two geometrical configurations. In Fig. S24, the experimental (B-integral-subtracted) values of $\Delta\varphi_7$ (red dots) and $\Delta\varphi_9$ (blue dots) from this study (the same data as in Fig. 4 of the main text) are compared to the reflection $\Delta\varphi_7$ (red dashed line) and $\Delta\varphi_9$ (blue

dashed line) results reported in Ref. [35], along with our theoretical simulations. The comparison is made within the $\Delta I/I_0 = \pm 0.21$ range, assuming a peak intensity of I_0 = 12 TWcm^{-2} inside the MgO sample (where at $\Delta I/I_0 = 0$, $I_1 = I_2 = 6$ TWcm^{-2}), as used in this work. The results clearly indicate that the dipole phase data obtained experimentally in this study's transmission geometry (corrected for the nonlinear effects) and the reflection measurements from Ref. [35] are consistent with each other and with the theoretical models. The dipole phase values for H7 and H9 are determined to be $\Delta\varphi_7$ = 1.48 $\pm$ 1.01 rad and $\Delta\varphi_9$ = 1.63 $\pm$ 1.25 rad in our work, while Lu *et al.* reported $\Delta\varphi_7$ = 0.97 rad (0.15*2π rad) and $\Delta\varphi_9$ = 1.07 rad (0.17*2π rad).
